# Do Birds Select Habitat or Food Resources? Nearctic-Neotropic Migrants in Northeastern Costa Rica

**DOI:** 10.1371/journal.pone.0086221

**Published:** 2014-01-28

**Authors:** Jared D. Wolfe, Matthew D. Johnson, C. John Ralph

**Affiliations:** 1 School of Renewable Natural Resources, Louisiana State University Agricultural Center and Louisiana State University, Baton Rouge, Louisiana, United States of America; 2 Department of Wildlife, Humboldt State University, Arcata, California, United States of America; 3 Redwood Sciences Laboratory, United States Department of Agriculture Forest Service, Arcata, California, United States of America; 4 Klamath Bird Observatory, Ashland, Oregon, United States of America; University of Regina, Canada

## Abstract

Nearctic-neotropic migrant birds need to replenish energy reserves during stopover periods to successfully complete their semiannual movements. In this study we used linear models to examine the habitat use of 11 migrant species in northeastern Costa Rica to better understand the influence of food and structural resources on the presence of birds during stopover periods. Our models indicated that frugivorous migrants primarily used food abundance, while insectivorous migrants chiefly used vegetation structure as cues for habitat use during stopover. In addition to habitat use models, we documented fruiting plant phenology and found a general relationship between migrant arrival and the timing of ripe fruit availability. Our results suggest that insectivorous migrants probably rely on structural features when using habitat because it may be inherently difficult to assess cryptic-arthropod availability during a short period of time in a novel habitat, such as stopover periods.

## Introduction

Mortalities accrued during migratory periods may regulate population growth of some bird species [1]–[3]. For example, Sillett and Holmes [2] estimated that 85% of apparent mortality occurred during migratory periods for populations of Black-throated Blue Warblers (*Setophaga caerulescens*) captured in New Hampshire, USA, and Jamaica. During their migratory periods, Nearctic-neotropic migrants (hereafter referred to as ‘migrants’) rely on stopover habitats that provide reliable food resources where birds can replenish their energy reserves necessary to successfully complete their semi-annual movements [4]–[5]. To date, studies have associated changes in migrant behavior, fat accumulation and stopover duration with habitat quality, thus, emphasizing the importance of assessing ecological value and conserving stopover areas for vulnerable migrant species [5]–[8]. In addition to conservation, examining relationships between stopover areas and habitat use can identify the behavioral adaptations migrants employ, such as diet switching from insects to fruit, to successfully use a diversity of habitats throughout temperate and tropical latitudes [9]–[11].

During migration many species use multiple cues to first select stopover areas and then decide whether to stay or leave [12]–[13]. Cues pertaining to initial stopover habitat selection and subsequent use are not necessarily exclusive and may depend heavily on predator avoidance and (or) food resource availability. Therefore, stopover habitat use may represent interactions between access to food and the vegetative cover necessary to avoid predators. To explore the influence of food and vegetative structure on migrant habitat use we simultaneously collected bird capture, food resource and structure data during fall migration in northeastern Costa Rica to test the following predictions: (1) If habitat use is influenced by migrant diet and spatial variation in resource supply, then high capture rates will correspond with sites rich in fruit and (or) insects; (2) If habitat use is influenced by selection for specific structural features (e.g., tree height, foliage density), then high capture rates will correspond with sites with specific, preferred vegetative characteristics.

## Methods

Bird banding stations were located near the village of Tortuguero on the northeast coast of Costa Rica, in Limon Province (Longitude: 83°31′7′′W Latitude: 10°33′51′′N). The study area is dominated by lowland, wet broadleaf tropical forest [14] that is dissected by canals and rivers that flow east into the Caribbean Sea. The area receives an average rainfall of >5 m per year [15], making it one of the wettest regions in the country. Tortuguero's wet season begins in mid to late April and continues through January. The wet season is interrupted by a short dry season during September. The long dry season tends to occur through February and March but precipitation is common even during this period [15]. Forest lands surrounding the village are protected by the 170,000 ha Tortuguero National Park and Barra del Colorado National Wildlife Refuge. We captured birds during fall migration from 1 September until 31 November 2008. Two stations were in mature forest approximately one kilometer west of the Caribbean Sea adjoining large wooded areas. One station was in mature forest bordered by coastal scrub and large wooded areas adjacent to the Caribbean Sea, and two were in a mixed habitat comprised of young forest and coastal scrub adjoining large wooded areas adjacent to the Caribbean Sea. Each capture station had 10–13 net sites (12×3 m, 36 mm mesh), totaling 56 net sites. All nets sites were strategically placed in a diversity of presumably suitable habitats to facilitate migrant and resident bird captures. Stations were at least two kilometers apart from each other. Net sites within each station were 40–70 m apart from each other; however, four of the 56 net sites were approximately 30 m from their nearest neighboring net site. One station was operated at least five times every 10 days, while the other four stations were operated at least once every 10 days. Stations were operated for six hours, starting 15 minutes before sunrise. All bird capture data has been archived through the Landbird Monitoring Network of the Americas (LaMNA).

Eleven species of migrants were chosen for study based on the diversity of dietary guilds they represent [16]–[20] and their historically high capture rates (Table 1). Humboldt State University's Institutional Animal Care and Use Committee (IACUC) committee specifically approved this study (07/08.W.42.A). Additionally, this study was lawfully conducted on private and public lands with the expressed permission from the following land-holders: the Sea Turtle Conservancy (for the CCC site), Tortuga Lodge (for the TORT site), Canadian Organization for Tropical Education & Rainforest Conservation (for the CANO site), and the Ministerio de Ambiente, Energía y Telecomunicaciones (for the PARQ and AERO sites). All necessary permits required to lawfully conduct our research were obtained from the United States Fish and Wildlife Service (permit #22030) and from the Ministerio de Ambiente, Energía y Telecomunicaciones (MINAE) of Costa Rica (permit # ACTo_GASP-PIN-02-2009).

**Table 1:** The degree of frugivory of the study species while in tropical latitudes; ‘+’ indicates moderate frugivory, ‘++’ indicates high frugivory, ‘−’ indicates little to no frugivory.

Target Species	Fall	Winter	Spring	Guild Description	Number of Captures
Acadian Flycatcher (*Empidonax alnorum*)	**++**	**++**	**++**	Frugivore/Aerial insectivore	51
Canada Warbler (*Cardellina canadensis*)	**+**	**+**	**+**	Gleaner/Aerial insectivore/Frugivore	32
Eastern Wood-Pewee (*Contopus virens*)	**−**	**−**	**−**	Aerial insectivore	41
Gray-cheeked Thrush (*Catharus minimus*)	**++**	**++**	**++**	Frugivore/Gleaner	102
Mourning Warbler (*Geothlypis philadelphia*)	**−**	**−**	**−**	Gleaner	23
Northern Waterthrush (*Parkesia noveboracensis*)	**−**	**−**	**+**	Gleaner	85
Prothonotary Warbler (*Protonotaria citrea*)	**++**	**−**	**−**	Gleaner/Frugivore	69
Red-eyed Vireo (*Vireo olivaceus*)	**+**	**+**	**+**	Frugivore/Gleaner/Aerial insectivore	20
Swainson's Thrush (*Catharus ustulatus*)	**++**	**++**	**++**	Frugivore/Gleaner	367
Traill's Flycatcher (*Empidonax alnorum/traillii*)	**++**	**++**	**++**	Frugivore/Aerial insectivore	100
Veery (*Catharus fuscescens*)	**++**	**++**	**++**	Frugivore/Gleaner	80

Guild description briefly describes the common foraging behavior of each species while in the neotropics. Number of captures refers to the total number of birds captured which were subsequently used to generate predictive linear models.

Once captured, droppings from study species were collected by placing each bird in a breathable paper bag until it defecated. Many migrants did not defecate while in captivity, or only passed white-uric acid with no discernable fruit or arthropods leading to a discrepancy between the number of captures and actual droppings sample size. Using a blunt-nosed probe, arthropod parts and fruit matter (pulp, seeds, etc.) were separated within a Petri dish. The percentage volume of fruit or arthropod matter within each dropping was estimated to the nearest 5% and averaged for each species. Seeds found within droppings were identified using a seed reference collection.

To quantify habitat structure, 15×15 m vegetation plots were centered at each net site [21]. Within each vegetation plot, the following measurements were taken: tree diameter at breast height (DBH), canopy closure, canopy height, soil moisture, tree density, vertical foliage density, and percentage ground cover (Figure S1). All structural measurements were taken during the month of January 2008; these data were analyzed as mean values per net site in subsequent models. Associations between habitat structure parameters were analyzed by subjecting habitat structure data (DBH, canopy closure, canopy height, soil moisture, tree density, vertical foliage density) to a principal components analysis. The first principal component value (PCA1) and total migrant capture rate were subsequently used in a simple linear regression to examine general patterns of migrant habitat use.

To quantify available fruit biomass, 5 (high) ×5 (wide) ×12 m (long) plots were centered within each vegetation plot, at each net site. Outside the plots, at least 50 individual fruit samples of the same species encountered within the plots were collected, seeds were extracted and the remaining fruit was weighed, yielding individual seedless wet-biomass values per fruit. Ripe fruit counts from plots were associated with biomass values to yield total ripe-fruit biomass estimates for the entire plot. The same process was also applied to unripe fruit within plots to estimate unripe-fruit biomass. Grams of sugar were estimated within each plot to determine the influence of calorie-rich fruit on migrant habitat use during stopover. Using a portable refractometer, individual Brix percentages (grams of solids per 100 grams of solution) were calculated for each species of sampled fruit outside the plot. Total seedless ripe-fruit biomass for a given species was multiplied by its associated Brix percentage yielding an approximation of available grams of sugar per fruit. Estimates of available grams of sugar per fruit were generated for each species within a plot and then added together, yielding a crude estimate of total grams of sugar for the entire plot. Within each fruit plot, ripe seedless-fruit biomass, unripe seedless-fruit biomass and grams of sugar were calculated for four periods during 2009 fall migration (1–21 Sep., 22 Sep. –15 Oct., 16 Oct. –5 Nov. and 6–29 Nov.).

Sweep netting was used to estimate arthropod biomass. Sweep netting is a general arthropod sampling method, well suited to capturing a variety of arthropod prey. Unlike ‘sticky boards,’ ‘pit-falls,’ or ‘branch-clips,’ which tend to focus on sampling aerial, terrestrial, and sessile arthropods respectively [22]–[23], sweep-nets tend to sample both aerial and sessile (and therefore plant obligates) arthropod species. Arthropod abundance was quantified by creating four 3×3×3 m subplots located within each plot. Each subplot was sampled once, for five minutes, over the course of fall migration. During each visit, one of the subplots within the plot was visited on a rotational basis; this technique ensured that subplots were only visited once. Because sampling aerial and sessile arthropod communities with sweep-nets do not reveal actual arthropod availability for all birds; here, sweep net samples provide an index of general arthropod abundance for gleaning and aerial-sallying migrant species. Arthropods collected were measured, and identified to order. Orders were separated into two functional groups: (1) winged-arthropods (all winged arthropods in adult stages thereby excluding larva, nymphs, ants, and Coleoptera with fused elytra); (2) total arthropods (all arthropod orders combined). Arthropod masses were derived using previously published length-mass regressions for tropical arthropod taxa [24]-[25]. Arthropod samples were conducted in four periods (1–21 Sep, 22 Sep. –15 Oct., 16 ct. –5 Nov. and 6–29 Nov.). This process yielded four values for each plot, representing fruit, sugar and arthropod biomass at equal time intervals across fall migration. The four temporally-distinct fruit, sugar and arthropod biomass values were averaged across periods, providing a single fruit biomass, arthropod biomass, and sugar value (in grams) for each plot which were used as covariates within habitat use models.

We formulated 30 competitive linear models for each of the 11 migrant study species, where capture rates per 100 net h (net specific capture-rate for a given species with captures summed across the three month period) were used as response variables in multiple linear regression models. Recaptures only consisted of same-day recaptures, and were not included in the analysis. Independent variables were subjected to a pairs plot to identify multicollinearity among the variables; highly correlated variables were not used within the same model. Candidate model covariates were included based on previously described habitat preferences [26]–[27], analysis of migrant droppings collected, and ancillary foraging observations recorded during the study. Covariate interactions were included to account for probable multiplicative relationships between food and habitat and, as they pertain to omnivorous species, between fruit and insects. Model covariates included PCA1, habitat structure variables, fruit and arthropod biomass variables. Top models were selected by comparing corrected Akaike Information Criterion (AICc) weights and values [28]. Q-Q plots were visually assessed to ensure normality.

Competing models within two AICc values were averaged using unconditional shrinkage estimators [29]. Inferences based on model results were restricted to the top model or averaged models within two AICc values of the top model. To further explore associations between bird captures and explanatory variables we calculated *p*-values and 95% confidence intervals for covariates in each top or averaged model. All analyses were conducted in Program R [30].

## Results

Between 1 September and 31 November 2009, 1,096 migrant birds representing 36 species were captured and processed. Of these, 970 individuals were of the eleven study species (Table 1). Also during the course of our study, 425 resident tropical birds were captured (not including hummingbirds) potentially indicating stronger intraspecific competition among migrants relative to interspecific competition from resident birds for food resources during migration.

**Figure 1:**
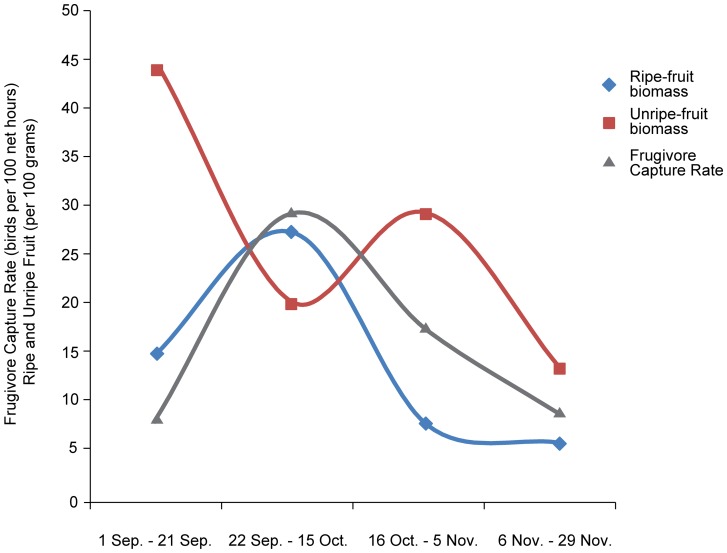
Frugivore capture rate (all study species except Northern Waterthrush, Eastern Wood-Pewee, Mourning Warbler, Canada Warbler) averaged across all net sites for each of the four sampling periods and associated summed seedless-ripe fruit biomass, across all vegetation plots for each of the four sampling periods in Tortuguero, Costa Rica, 2008.

Unripe fruit was most common at the beginning of fall migration (1 Sep –21 Sep), with 11.5 g of estimated seedless biomass per 10 m^3^. This value declined through the end of November (Figure 1). Ripe fruit was most common during the second period (22 Sep to 15 October), corresponding with peak frugivore capture rate, both then declined through the end of fall migration. The peak capture rate of the six species of frugivorous migrants (Acadian Flycatcher, Gray-cheeked Thrush, Prothonotary Warbler, Red-eyed Vireo, Swainson's Thrush, Traill's Flycatcher and Veery) at 0.24 frugivorous migrants per net h, was synchronized with maximum availability of ripe fruit (6.5 g of total estimated seedless ripe-fruit biomass per 100 m^3^). No clear temporal pattern was detected in the sampled arthropod community (Figure S2).

**Figure 2:**
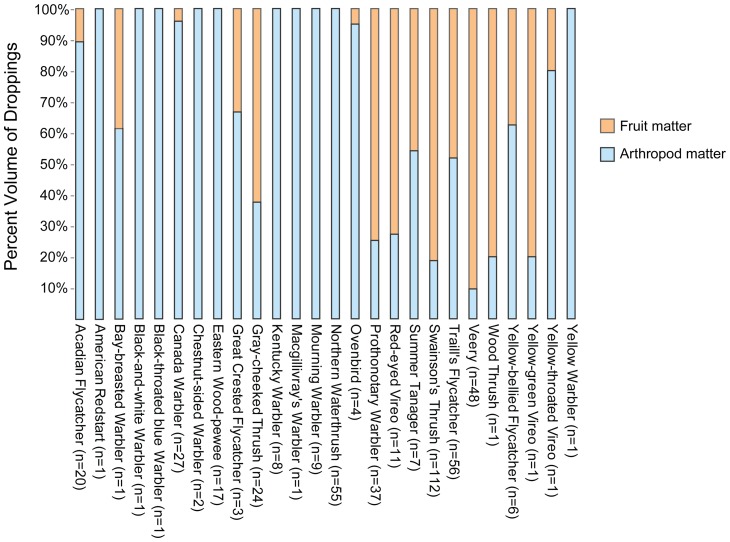
Averaged volumetric estimates of dropping contents and associated sample sizes for migrant birds captured in Tortuguero, Costa Rica, during 1 sept. –31 Nov. 2008.

In total, 15 of the 16 frugivorous study species had droppings containing seeds of *Conostegia xalapensis* (family: Melastomataceae), a common shrub found on the edges of rivers, streams and within coastal scrub (Table 2). Surprisingly, droppings of Prothonotary Warbler, usually considered an insectivore, were comprised mostly of fruit (Figure 2), and only of *Conostegia xalapensis* (Table 2).

**Table 2:** Percent occurrence of fruit identified.

	*Anthirium* spp.	*Citharexylum caudatum*	*Clidemia* spp.	*Conostegia xalapensis*	*Geonoma* spp.	*Palicourea guianensis*	*Psychotria brachiata*	*Psychotria glomerulata*	*Psychotria grandis*
Acadian Flycatcher (*Empidonax virescens*, *n* = 10)				10					
Bay-breasted Warbler (*Setophaga castanea*, *n* = 1)				n/a					
Canada Warbler (*Cardellina canadensis*, *n* = 27)				4					4
Great-Crested Flycatcher (*Myiarchus crinitus*, *n* = 3)				33					
Gray-cheeked Thrush (*Catharus minimus*, *n* = 24)		9		35		9			4
Ovenbird (*Seiurus aurocapilla*, *n* = 4)				25					
Prothonotary Warbler (*Protonotaria citrea*, *n* = 37)				78					
Red-eyed Vireo (*Vireo olivaceus*, *n* = 11)				55		9			
Summer Tanager (*Piranga rubra*, *n* = 7)	5	1		43		10	2	1	2
Traill's Flycatcher (*Empidonax alnorum/traillii*, *n* = 56)	2			41		5			
Veery (*Catharus fuscescens*, *n* = 48)			2	26	2	21	2		
Wood Thrush (*Hylocichla mustelina*, *n* = 1)							n/a		
Yellow-bellied Flycatcher (*Empidonax flaviventris*, *n* = 6)				17		17			
Yellow-green Vireo (*Vireo flavoviridis*, *n* = 1)				n/a					
Yellow-throated Vireo (*Vireo flavifrons*, *n* = 1)				n/a					

Column represents fruit taxa identified and rows represent bird species and droppings sample sizes collected in Tortuguero, Costa Rica, 1 September –31 November, 2008. Percent occurrence was not calculated for a species with 3 or fewer dropping samples (represented by n/a).

**Figure 3:**
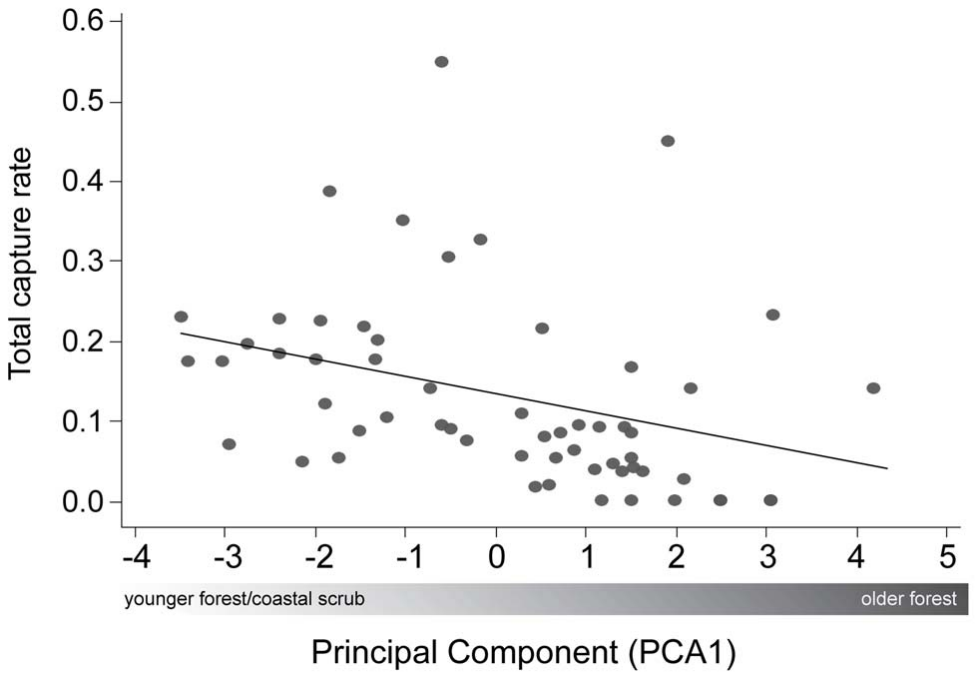
Linear regression between the first principal component (PCA1) and hourly capture rate of all study species (*P* = 0.013, adj. *R* ^2^ = 0.09). Positive PCA1 values are associated with mature forest habitat types. Tortuguero, Costa Rica, 2008.

We first characterized habitat by conducting a principal components analysis of structure data at each mist net site (Table 3). The first component (PCA1) included 41 percent of the total variance, while the second principal component added another 17 percent to the total variance explained. Values of the first component were positively correlated with older forest characteristics (higher DBH, taller forest, more canopy closure, higher soil moisture, and less understory foliage density; see Table 3). We found a negative correlation between total migrant capture rate and PCA1, indicating that fewer migrants were captured in older forests (*P* = 0.013, adj. *R* ^2^ = 0.09; Figure 3).

**Table 3:** Principal Component Analysis results summary of eight habitat and vegetation variables.

			Component				
			PCA1	PCA2	PCA3	PCA4	PCA5
Percentage of accounted for			0.41	0.17	0.15	0.10	0.05
Cumulative percentage of total variance accounted for			0.41	0.59	0.74	0.84	0.89
Correlations to original variables	DBH		0.42	−0.20	0.29	–0.08	0.53
	Tree Density		–0.14	0.12	–0.69	–0.61	0.21
	Canopy Height		0.51	–0.03	–0.06	–0.07	–0.01
	Canopy Closure		0.45	0.29	0.04	–0.05	0.27
	Percent Soil Moisture		0.43	0.01	–0.31	–0.06	–0.07
	Foliage Density 0–3m		–0.32	0.19	0.47	–0.49	0.40
	Foliage Density 3–15m		–0.13	0.65	–0.21	0.54	0.40
	Foliage Density >15m		0.19	0.64	0.27	–0.29	–0.53

Tortuguero, Costa Rica, 2008. Note that quantities ending in zero were truncated.

Only Northern Waterthrush yielded an uninformative model characterized by a competitive null model (within two AICc values), covariates with insignificant *p*-values (>0.05) and 95% confidence intervals that overlap ‘0’ (Table S1, S2, S3, S4, S5, S6, S7, S8, S9, S10, S11, S12). Habitat use for eight predominantly frugivorous migrants had fruit and (or) sugar within their respective top model or averaged model. In addition to positive correlations with ripe fruit, Veery and Gray-cheeked Thrush also had positive correlations with DBH, while Swainson's Thrush yielded a positive correlation with PCA1, possibly indicating a preference for relatively mature forest structure. By contrast, top models for three predominantly insectivorous migrants, Canada Warbler, Mourning Warbler and Eastern Wood-Pewee, emphasized structural characteristics and no food covariates (arthropod biomass; Table 4).

**Table 4:** Top or averaged linear models (within 2 AICc values of the top model) and associated null models for comparative purposes for 11 migrant species captured in Tortuguero, Costa Rica.

Species	Model	ΔAICc	w_i_	K
Acadian Flycatcher ^†^	**ripe fruit^+^**, sugar^+^, **PCA^−^**	-	0.22	5
	*null*	15.49	0.00	2
Canada Warbler	**foliage density 0–3m^+^**, **foliage density 3–15m^+^**	-	0.43	4
	*null*	12.11	0.00	2
Eastern Wood-Pewee	**PCA^−^**	-	0.36	3
	*null*	12.51	0.00	2
Grey-cheeked Thrush	ripe fruit**^+^**, **DBH^+^**	-	0.38	4
	*null*	3.97	0.05	2
Mourning Warbler	canopy closure^−^, **foliage density 0–3m^−^**, foliage density 0–3m*canopy closure^+^	-	0.81	5
	*null*	18.53	0.00	2
Northern Waterthrush^†^	arthropod total^+^, canopy height^−^, canopy closure^+^, foliage density 3–15m^+^, foliage density 0–3m^−^, DBH^−^, foliage density 0–3m*canopy closure^−^	-	0.08	9
	*null*	1.40	0.04	2
Prothonotary Warbler	sugar^+^, PCA^−^, sugar*PCA^−^	-	0.53	5
	*null*	16.15	0.00	2
Red-eyed Vireo^†^	foliage density 0–3m^+^, **ripe fruit^−^**	-	0.21	4
	*null*	5.73	0.01	2
Swainson's Thrush^†^	**ripe fruit^+^**, arthropod total^+^, **sugar^+^**, PCA1^+^, PCA1*ripe fruit^−^	-	0.23	7
	*null*	48.32	0.00	2
Traill's Flycatcher^†^	arthropod winged^+^, **PCA1^−^**, sugar^+^	-	0.26	5
	*null*	9.66	0.00	2
Veery^†^	ripe fruit, DBH, tree density, arthropod total	-	0.39	6
	*null*	7.13	0.01	2

Statistics include AICc differences between top-model and null model (ΔAICc), AICc weight (w_i_) and model parameter number (K). † indicates an averaged model; **bold** indicates that a covariate is statistically significant; +/− indicates positive or negative correlation with capture rate.

Top models for migrant *Empidonax* species contained sugar and (or) fruit (Table 4) and negative correlations with PCA1 (Table 4). The two top models for Red-eyed Vireo were within two AICc values of each other and were subsequently averaged (Table S8) yielding a positive correlation with ripe fruit (Table 4). The top model for Prothonotary Warbler contained a negative interaction between sugar and PCA1 (Table 4).

## Discussion

Resources influence habitat use of migrating landbirds in temperate North America [31] and during their over-wintering periods [32]–[33]. Our results suggest that food and (or) structural attributes influenced habitat use by 10 of the 11 migrant study species during stopover along the Caribbean coastal plain of Costa Rica.

We found that top models for frugivorous migrant species included fruit and (or) sugar predictor variables. Conversely, top or averaged models for insectivorous migrants (Canada Warbler, Mourning Warbler and Eastern Wood-Pewee) contained only structural characteristics, not food (arthropod biomass). Our results suggest that insectivorous migrants may use habitat favorable to their specific foraging behavior by means of vegetative cues, rather than directly assessing arthropod availability during stopover. Alternatively, the precision of our estimates of arthropod availability may have been insufficient to reveal correlations.

More specifically, Canada Warbler had a top model that included denser foliage in two lower canopy layers, indicative of a more complex understory commonly found in younger secondary forest and coastal scrub. The top model for Mourning Warbler yielded negative correlations with canopy closure and low-level foliage less than 3 m indicative of disturbed areas such as semi-open undergrowth along the Caribbean shore; previous assessments of Mourning Warblers wintering habitats found similar results [34]–[35]. Eastern Wood-Pewee had a top model negatively correlated with PCA1, representative of younger forest types. Previous studies have also documented migratory Eastern Wood-Pewees using younger forest types in Costa Rica [35]–[36]. Interestingly, results for Grey-cheeked Thrush, Swainson's Thrush and Veery indicated a preference for mature forested habitats, relative to young forest and scrub, with increased fruit availability.

Differences between insectivorous species using structural characteristics as a cue, relative to frugivorous species directly using fruit resources as a cue, may be explained evolutionarily. Fruiting bodies are often brightly colored to attract potential seed dispersers [37]. Conversely, to avoid predation, forest-dwelling arthropod species are often cryptic [38]. Because of their cryptic nature, it may be inherently difficult for an insectivore to assess availability of arthropods in novel habitats. Therefore, relying on proximate structural cues appropriate for a particular foraging strategy may maximize resource acquisition during stopover in unfamiliar surroundings.

Sugar was in the top or averaged model of four study species indicating a potential preference for habitats with sugar-rich fruit. However, multicollinearity between grams of sugar and fruit biomass in the averaged models makes such inferences difficult to substantiate. The prevalence of sugar and (or) fruit in the top or averaged models of six study species emphasizes the strong influence of fruit distribution and caloric intake on migrant habitat use during fall migration.

Migrant birds may avoid predation by selecting habitat conducive to predator avoidance such as increased foliage density [39] which was associated with understories in younger forest types (Figure S3, S4, S5, S6). Here, eight of the 11 study species had top or averaged models with vegetative attributes associated with younger forest types. Selecting habitat with increased foliage density, such as the understory of young forests (Figure S3, S4, S5, S6) may reduce predation risk; however, such inferences are difficult to evaluate because fruit abundance was also found to be greater in younger forest types (Figure S7). Younger forest types may present frugivorous migrant species with two attractive attributes: increased fruit resources and enhanced predator avoidance. This assertion is supported by PCA1 and results of the linear regression of total migrant capture rates, indicating that migrants were captured more commonly in secondary forest and coastal scrub, relative to older forests (Figure 3). Our finding that more migrants are captured in younger forests, coupled with wide-spread type conversion from primary to secondary forests in tropical latitudes [40], suggests that many migrant songbird species might not be limited by the availability of stopover habitat in Central America.

Peak migration coincided with the maximum availability of ripe fruit (Figure 1). This pattern may be explained in four ways: (1) coincidental synchrony between unrelated events; (2) the mutualistic relationship between seed-dispersing migrant frugivores and amount of fruit produced by plants; (3) fruiting plants evolved a phenology associated with day length, solar exposure and precipitation, and migrants distribute themselves spatially and temporally relative to available fruit resources; and (4) both migrants and fruiting plants are responding phenologically to both photoperiod and weather in identical ways. If peak migration coincided with high availability of ripe fruit due to a mutualistic relationship between migrant frugivores and fruiting plants, then deviation from the timing of peak fruit masts or from migratory bird passage might negatively impact both plant and migrant communities. For example, Wolfe and Ralph [6] documented correlations between poor body condition of frugivorous migrants and dry El Niño periods in northeastern Costa Rica, possibly due to climatically-induced depressions of fruit production. This suggests there is a complex “balance” between climate, fruit availability, habitat use, and fat deposition. However, in an old world Palearctic system there is a disconnects between plant phenology and migrant arrival where fruiting plants responded to climatic patterns and migrants responded to photoperiod [41].

Here, we present insights into migrant habitat use, and relationships between fruit availability and migrant arrival time during stopover in northeastern Costa Rica. We suggest future research focus on determining the influence of climate on plant phenology and migrant birds during stopover in tropical latitudes. Identifying mechanisms responsible for variation in migrant food availability (e.g. climate, migrant arrival times, or both) will allow managers to predict, and potentially mitigate, future migrant conservation challenges.

## Supporting Information

Figure S1 **Plots were centered around each net site and were used to quantify structural characteristics, fruit and arthropod biomass.** Measurements at each star included: vertical foliage density, percent canopy closure and percent soil moisture Measurements within the entire 15×15 m vegetation plot included: tree diameter at breast height (DBH), tree density and canopy height.
(TIF)

Figure S2 **Insectivore capture rate per 25 net h (all study species except Grey-cheeked Thrush, Prothonotary Warbler, Swainson's Thrush, Red-eyed Vireo, Veery, Wood Thrush, Yellow-green Vireo) averaged across all net sites for each of the four sampling periods and associated summed arthropod biomass standardized by grams per 100 g, across all vegetation plots for each of the four sampling periods in Tortuguero, Costa Rica, 2008.**
(TIF)

Figure S3 **Box plot, with sample sizes (n =  number of sample locations; 4 sample locations within each vegetation plot), of vertical foliage density at primary forest net sites.** Heights are separated into three categories: 0–5 m, 3–15 m and >15 m.
(TIF)

Figure S4 **Box plot, with sample sizes (n =  mber of sample locations; 4 sample locations within each vegetation plot), of vertical foliage density at coastal scrub net sites.** Heights are separated into three categories: 0–5 m, 3–15 m and >15 m.
(TIF)

Figure S5 **Box plot, with sample sizes (n =  umber of sample locations; 4 sample locations within each vegetation plot), of vertical foliage density at secondary forest net sites.** Heights are separated into three categories: 0–5 m, 3–15 m and >15 m.
(TIF)

Figure S6 **Box plot, with sample sizes of percent soil moisture averaged across all four sampling periods.**
(TIF)

Figure S7 **Box plot, with sample sizes (n =  umber of 5×5×12 m fruit plots), of seedless ripe fruit biomass (g) averaged.**
(TIF)

Table S1 **Acadian Flycatcher habitat use model results.** Birds were captured in Tortuguero, Costa Rica, during the 2008 fall migration. The response variable is birds captured per 100 net hours.
(DOCX)

Table S2 **Canada Warbler habitat use model results.** Birds were captured in Tortuguero, Costa Rica, during the 2008 fall migration. The response variable is birds captured per 100 net hours.
(DOCX)

Table S3 **Eastern Wood-Pewee habitat use model results.** Birds were captured in Tortuguero, Costa Rica, during the 2008 fall migration. The response variable is birds captured per 100 net hours.
(DOCX)

Table S4 **Gray-cheeked Thrush habitat use model results.** Birds were captured in Tortuguero, Costa Rica, during the 2008 fall migration. The response variable is birds captured per 100 net hours.
(DOCX)

Table S5 **Mourning Warbler habitat use model results.** Birds were captured in Tortuguero, Costa Rica, during the 2008 fall migration. The response variable is birds captured per 100 net hours.
(DOCX)

Table S6 **Northern Waterthrush habitat use model results.** Birds were captured in Tortuguero, Costa Rica, during the 2008 fall migration. The response variable is birds captured per 100 net hours.
(DOCX)

Table S7 **Prothonotary Warbler habitat use model results.** Birds were captured in Tortuguero, Costa Rica, during the 2008 fall migration. The response variable is birds captured per 100 net hours.
(DOCX)

Table S8 **Red-eyed Vireo habitat use model results.** Birds were captured in Tortuguero, Costa Rica, during the 2008 fall migration. The response variable is birds captured per 100 net hours.
(DOCX)

Table S9 **Swainson's Thrush habitat use model results.** Birds were captured in Tortuguero, Costa Rica, during the 2008 fall migration. The response variable is birds captured per 100 net hours.
(DOCX)

Table S10 **Traill's Flycatcher habitat use model results.** Birds were captured in Tortuguero, Costa Rica, during the 2008 fall migration. The response variable is birds captured per 100 net hours.
(DOCX)

Table S11 **Veery habitat use model results.** Birds were captured in Tortuguero, Costa Rica, during the 2008 fall migration. The response variable is birds captured per 100 net hours.
(DOCX)

Table S12 **Linear model variables for 11 migrant species captured in Tortuguero, Costa Rica, and their associated beta estimates, standard errors and 95% confidence intervals for best or averaged models of each study species.** Asterisk indicates an averaged model.
(DOCX)
