# Supplementary figures and images for: Do Birds Select Habitat or Food Resources? Nearctic-Neotropic Migrants in Northeastern Costa Rica
Source: PLoS One. 2014 Jan 28;9(1):e86221. doi: 10.1371/journal.pone.0086221 (PMC3904878; doi:10.1371/journal.pone.0086221)

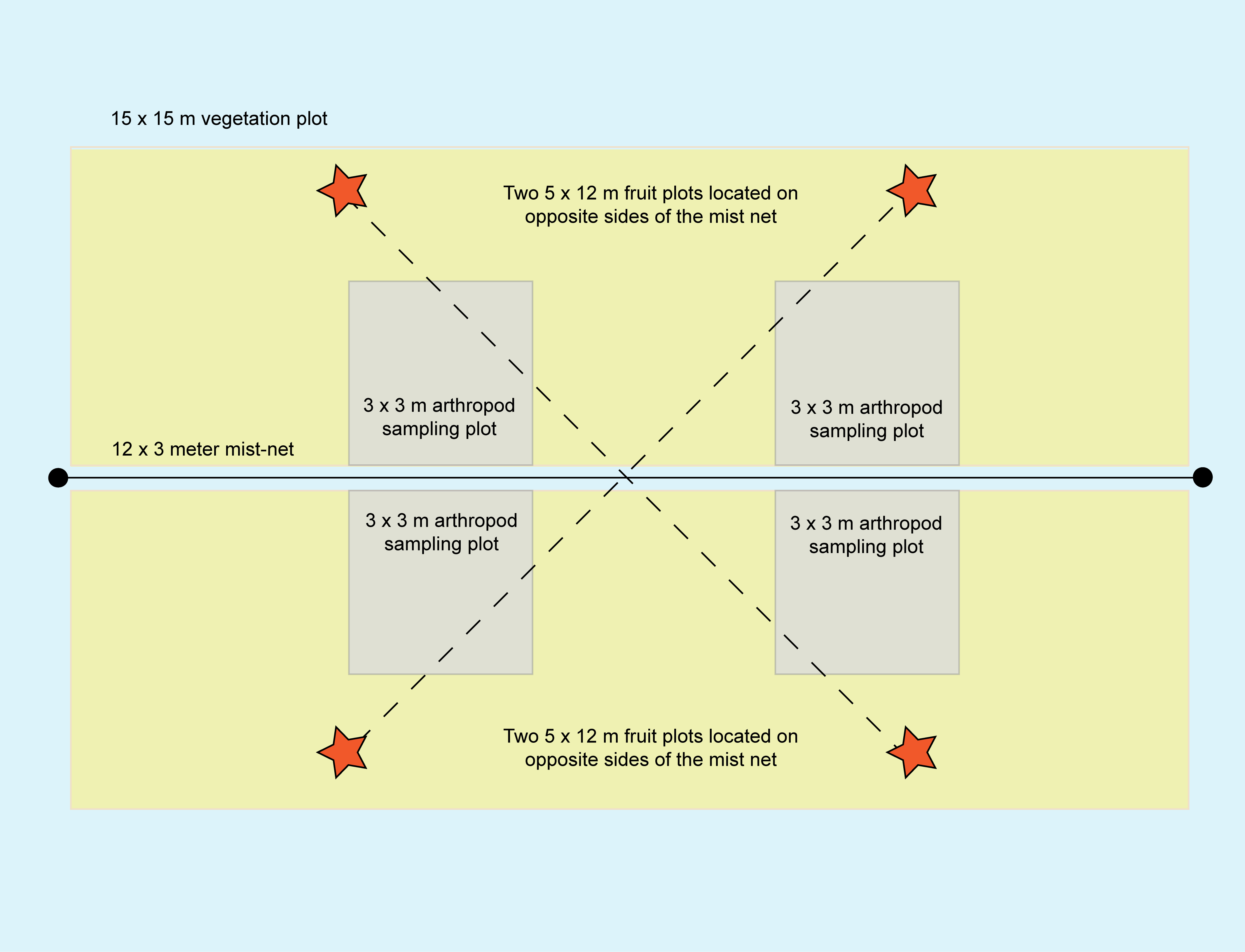

Supplement: Figure S1 — Plots were centered around each net site and were used to quantify structural characteristics, fruit and arthropod biomass. Measurements at each star included: vertical foliage density, percent canopy closure and percent soil moisture Measurements within the entire 15×15 m vegetation plot included: tree diameter at breast height (DBH), tree density and canopy height. (TIF) [file pone.0086221.s001.tif]

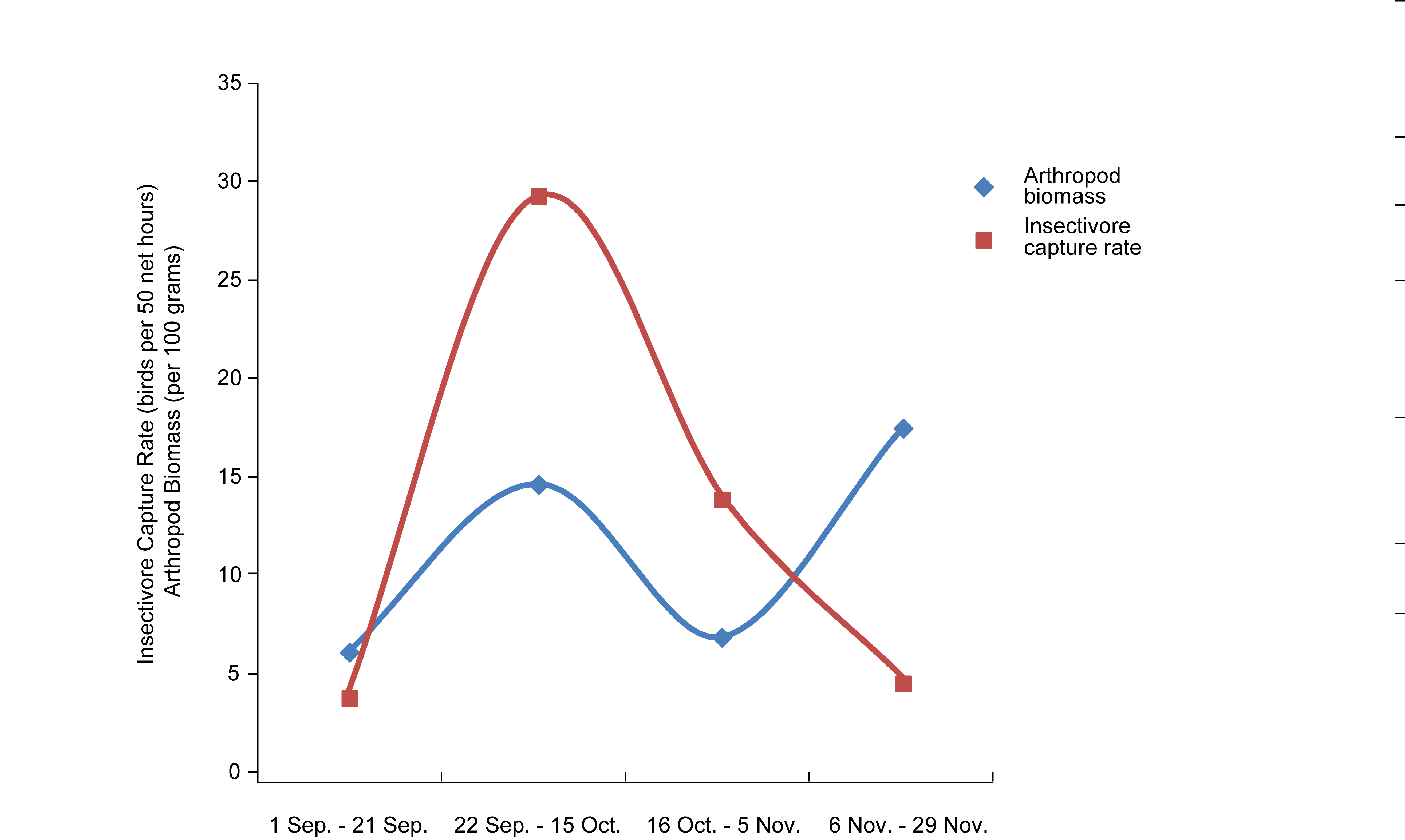

Supplement: Figure S2 — Insectivore capture rate per 25 net h (all study species except Grey-cheeked Thrush, Prothonotary Warbler, Swainson's Thrush, Red-eyed Vireo, Veery, Wood Thrush, Yellow-green Vireo) averaged across all net sites for each of the four sampling periods and associated summed arthropod biomass standardized by grams per 100 g, across all vegetation plots for each of the four sampling periods in Tortuguero, Costa Rica, 2008. (TIF) [file pone.0086221.s002.tif]

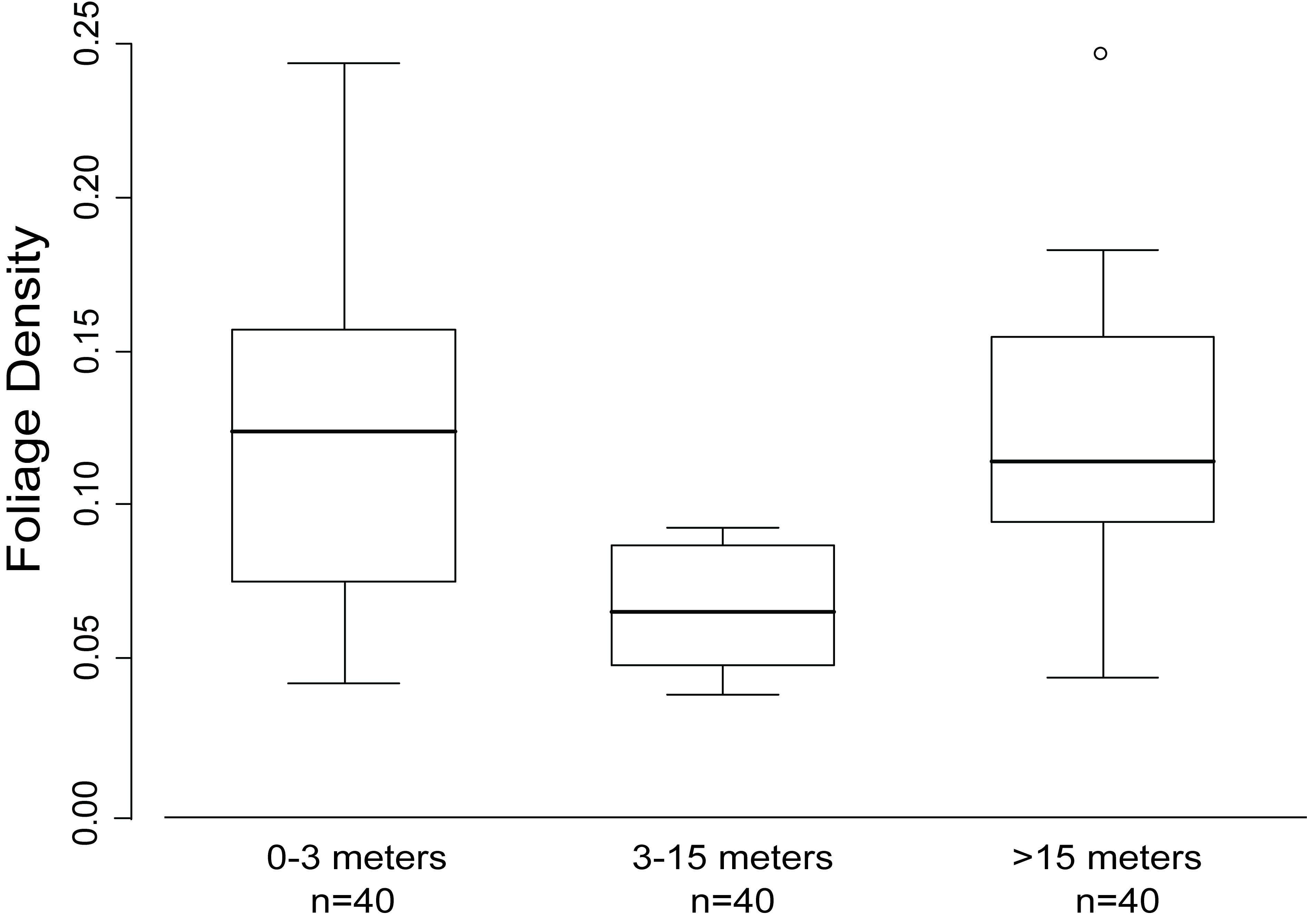

Supplement: Figure S3 — Box plot, with sample sizes (n = number of sample locations; 4 sample locations within each vegetation plot), of vertical foliage density at primary forest net sites. Heights are separated into three categories: 0–5 m, 3–15 m and >15 m. (TIF) [file pone.0086221.s003.tif]

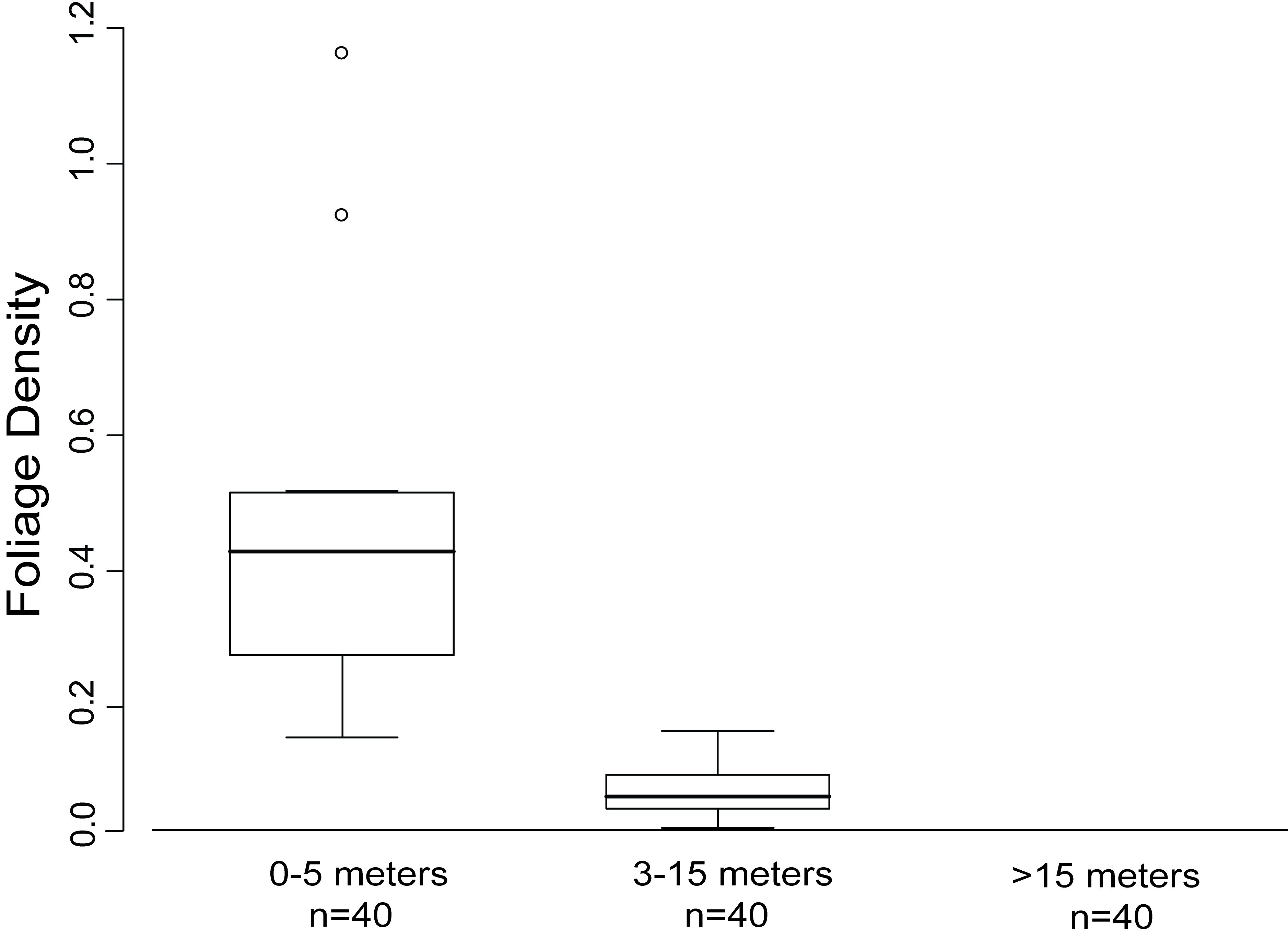

Supplement: Figure S4 — Box plot, with sample sizes (n = mber of sample locations; 4 sample locations within each vegetation plot), of vertical foliage density at coastal scrub net sites. Heights are separated into three categories: 0–5 m, 3–15 m and >15 m. (TIF) [file pone.0086221.s004.tif]

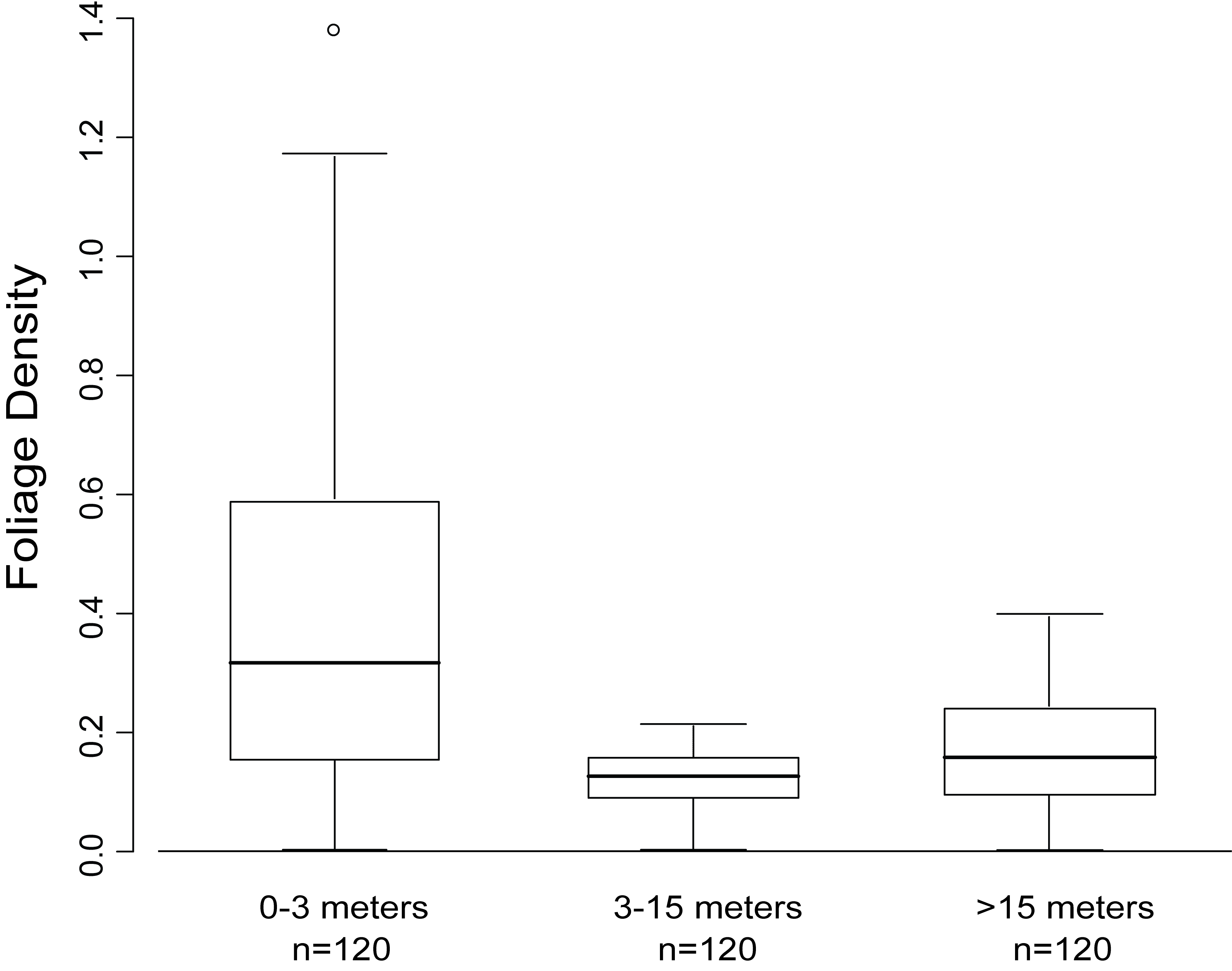

Supplement: Figure S5 — Box plot, with sample sizes (n = umber of sample locations; 4 sample locations within each vegetation plot), of vertical foliage density at secondary forest net sites. Heights are separated into three categories: 0–5 m, 3–15 m and >15 m. (TIF) [file pone.0086221.s005.tif]

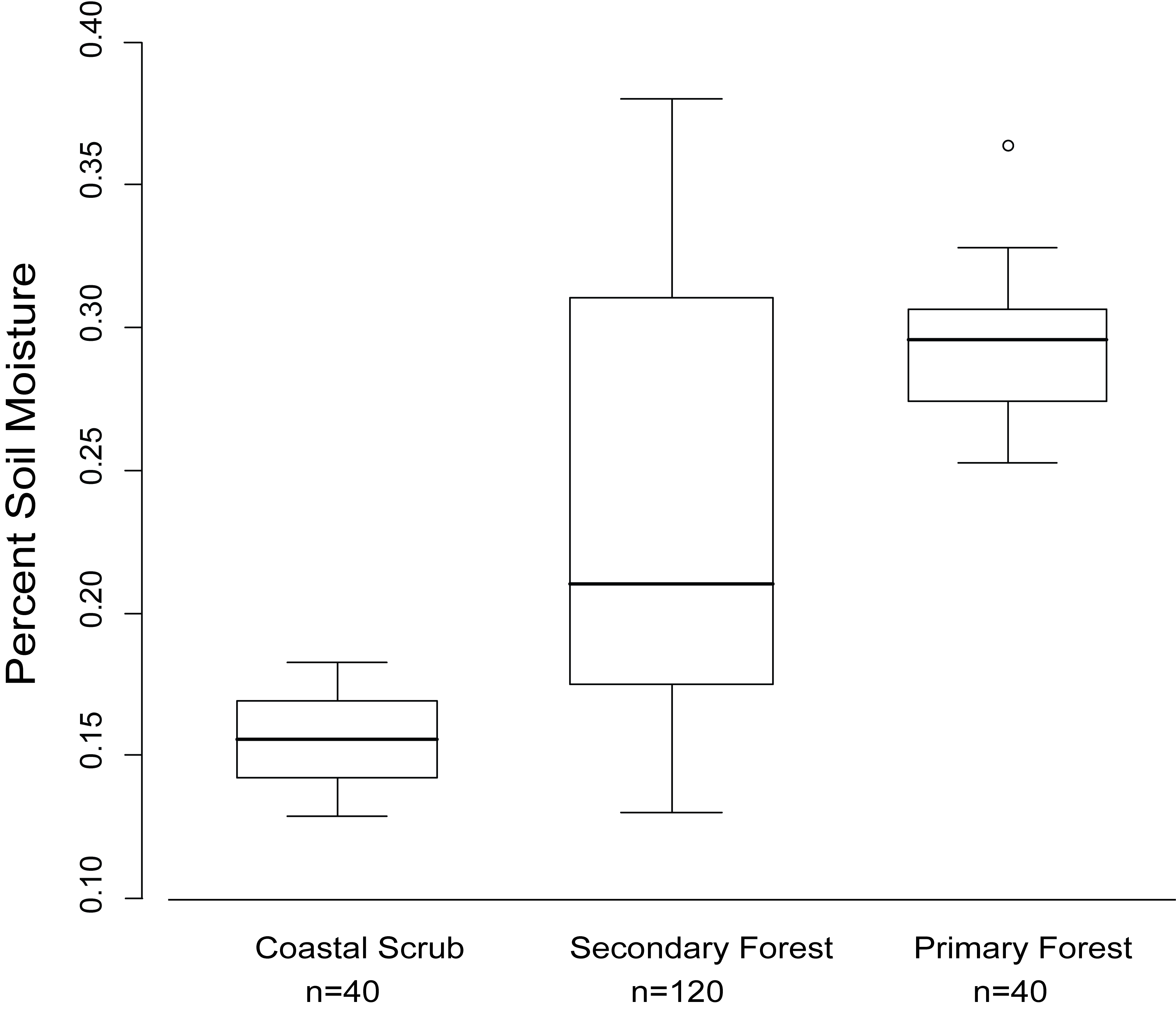

Supplement: Figure S6 — Box plot, with sample sizes of percent soil moisture averaged across all four sampling periods. (TIF) [file pone.0086221.s006.tif]

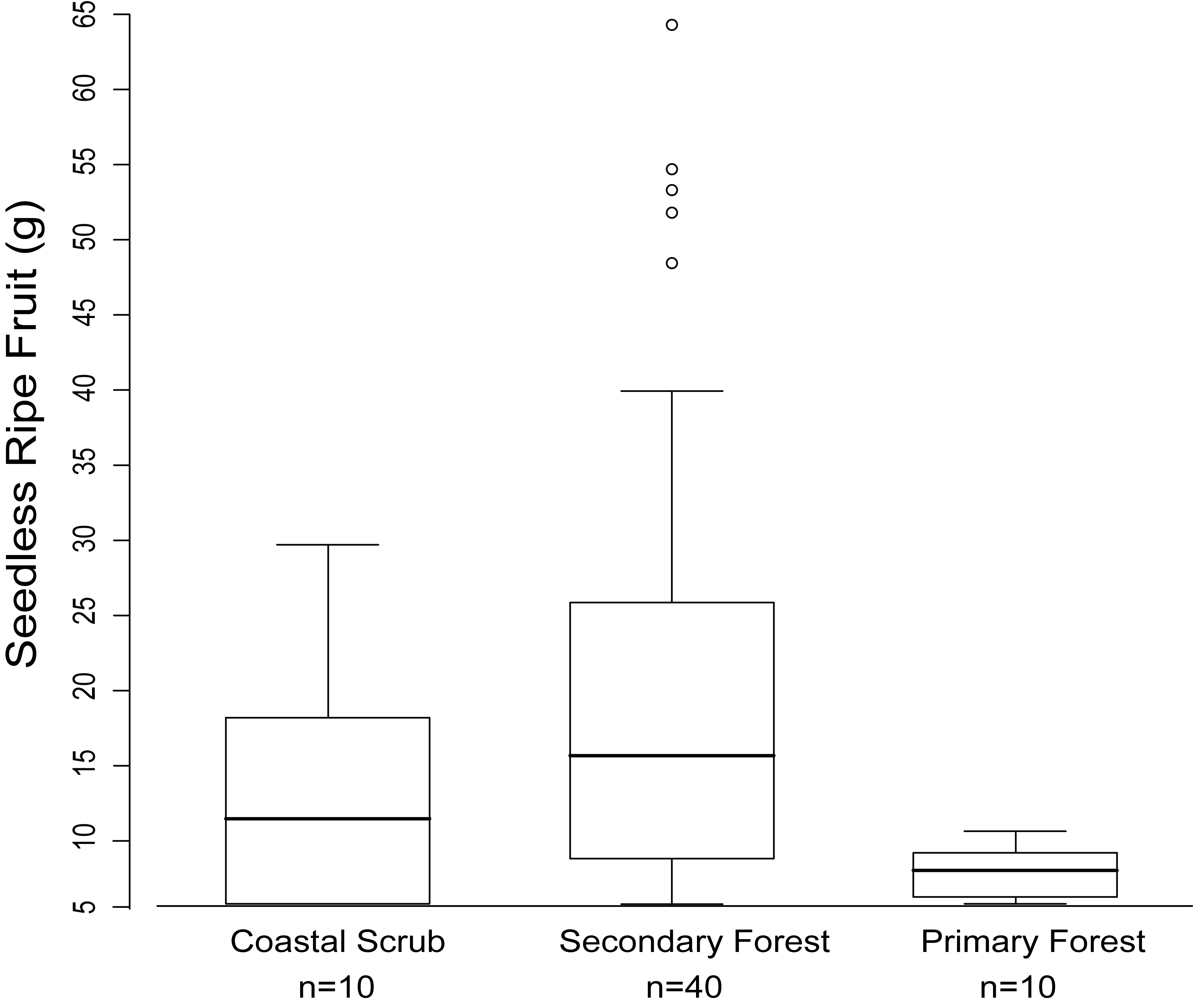

Supplement: Figure S7 — Box plot, with sample sizes (n = umber of 5×5×12 m fruit plots), of seedless ripe fruit biomass (g) averaged. (TIF) [file pone.0086221.s007.tif]
