# Supplementary material for: Do Birds Select Habitat or Food Resources? Nearctic-Neotropic Migrants in Northeastern Costa Rica
Source: PLoS One. 2014 Jan 28;9(1):e86221. doi: 10.1371/journal.pone.0086221 (PMC3904878; doi:10.1371/journal.pone.0086221)
Supplement: Table S1 — Acadian Flycatcher habitat use model results. Birds were captured in Tortuguero, Costa Rica, during the 2008 fall migration. The response variable is birds captured per 100 net hours. (DOCX) [file pone.0086221.s008.docx]

Table S1.

| Model | *p*-value | adj. *R^2^* | ΔAICc | w_i_ | K |
| --- | --- | --- | --- | --- | --- |
| ripe fruit+PCA | 0.0001 | 0.27 | 0.00 | 0.22 | 4 |
| sugar+PCA | 0.0001 | 0.27 | 0.24 | 0.20 | 4 |
| arthropod total+ripe fruit+PCA | 0.0003 | 0.26 | 2.16 | 0.08 | 5 |
| foliage density 0-3m | 0.0001 | 0.23 | 2.18 | 0.08 | 3 |
| PCA | 0.0001 | 0.23 | 2.39 | 0.07 | 3 |
| PCA+sugar+PCA*sugar | 0.0003 | 0.26 | 2.60 | 0.06 | 5 |
| ripe fruit+canopy closure+foliage density 0-3m | 0.0004 | 0.25 | 3.12 | 0.05 | 5 |
| sugar+canopy closure+foliage density 0-3m | 0.0004 | 0.25 | 3.13 | 0.05 | 5 |
| arthropod total*ripe fruit+arthropod total+ripe fruit+canopy closure+foliage density 0-3m | 0.0006 | 0.28 | 3.80 | 0.03 | 7 |
|  |  |  |  |  |  |
| canopy height+canopy closure+foliage density 0-3m | 0.0006 | 0.24 | 3.82 | 0.03 | 5 |
| foliage density 0-3m+foliage density 3-15m | 0.0005 | 0.22 | 3.93 | 0.03 | 4 |
| canopy closure+foliage density 0-3m+foliage density 3-15m | 0.0007 | 0.24 | 4.24 | 0.03 | 5 |
| arthropod winged+PCA | 0.0007 | 0.21 | 4.71 | 0.02 | 4 |
| sugar+canopy height+canopy closure+foliage density 0-3m | 0.0009 | 0.25 | 4.86 | 0.02 | 6 |
| canopy closure+foliage density 0-3m+foliage density 3-15m+canopy height | 0.0015 | 0.23 | 6.10 | 0.01 | 6 |

| Model | *p*-value | adj. *R^2^* | ΔAICc | w_i_ | K |
| --- | --- | --- | --- | --- | --- |
| canopy height | 0.0009 | 0.17 | 6.19 | 0.01 | 3 |
| DBH*canopy closure+foliage density 0-3m+DBH+canopy closure | 0.0017 | 0.23 | 6.38 | 0.01 | 6 |
| canopy height+canopy closure | 0.0043 | 0.16 | 8.51 | 0.00 | 4 |
| arthropod total*sugar+arthropod total+sugar | 0.0097 | 0.15 | 10.28 | 0.00 | 5 |
| sugar | 0.0168 | 0.08 | 11.74 | 0.00 | 3 |
| arthropod total*ripe fruit+arthropod total+ripe fruit | 0.0214 | 0.12 | 12.12 | 0.00 | 5 |
| ripe fruit | 0.0236 | 0.07 | 12.35 | 0.00 | 3 |
| arthropod winged+sugar | 0.0532 | 0.07 | 13.84 | 0.00 | 4 |
| arthropod total+sugar | 0.0547 | 0.07 | 13.90 | 0.00 | 4 |
| arthropod winged+ripe fruit | 0.0719 | 0.06 | 14.48 | 0.00 | 4 |
| arthropod total+ripe fruit | 0.0739 | 0.06 | 14.54 | 0.00 | 4 |
| null | n/a | n/a | 15.49 | 0.00 | 2 |
| tree density | 0.7385 | 0.00 | 17.60 | 0.00 | 3 |
| arthropod total | 0.9723 | 0.00 | 17.72 | 0.00 | 3 |
| arthropod winged | 0.9908 | 0.00 | 17.72 | 0.00 | 3 |
