# Supplementary material for: Do Birds Select Habitat or Food Resources? Nearctic-Neotropic Migrants in Northeastern Costa Rica
Source: PLoS One. 2014 Jan 28;9(1):e86221. doi: 10.1371/journal.pone.0086221 (PMC3904878; doi:10.1371/journal.pone.0086221)
Supplement: Table S2 — Canada Warbler habitat use model results. Birds were captured in Tortuguero, Costa Rica, during the 2008 fall migration. The response variable is birds captured per 100 net hours. (DOCX) [file pone.0086221.s009.docx]

Table S2.

| Model | *p*-value | adj. *R^2^* | ΔAICc | w_i_ | K |
| --- | --- | --- | --- | --- | --- |
| foliage density 0-3m+foliage density 3-15m | 0.0004 | 0.23 | 0.00 | 0.43 | 4 |
| canopy closure+foliage density 0-3m+foliage density 3-15m | 0.0014 | 0.21 | 2.41 | 0.13 | 5 |
| foliage density 0-3m+foliage density 3-15m+DBH | 0.0014 | 0.21 | 2.42 | 0.13 | 5 |
| foliage density 0-3m | 0.0012 | 0.16 | 3.32 | 0.08 | 3 |
| foliage density 0-3m+tree density | 0.0024 | 0.17 | 3.93 | 0.06 | 4 |
| DBH+foliage density 0-3m | 0.0041 | 0.16 | 5.03 | 0.03 | 4 |
| canopy closure+foliage density 0-3m+canopy_height | 0.0049 | 0.17 | 5.34 | 0.03 | 5 |
| arthropod total+foliage density 0-3m | 0.0052 | 0.15 | 5.55 | 0.03 | 4 |
| canopy closure+foliage density 0-3m | 0.0053 | 0.15 | 5.59 | 0.03 | 4 |
| canopy closure+foliage density 0-3m+foliage density 0-3m*canopy closure | 0.0136 | 0.14 | 7.69 | 0.01 | 5 |
| canopy_height | 0.0134 | 0.09 | 7.95 | 0.01 | 3 |
| canopy_height+canopy closure | 0.0166 | 0.11 | 8.01 | 0.01 | 4 |
| foliage density 3-15m | 0.0186 | 0.08 | 8.55 | 0.01 | 3 |
| PCA | 0.0226 | 0.08 | 8.90 | 0.01 | 3 |
| arthropod total+canopy_height | 0.0480 | 0.07 | 10.25 | 0.00 | 4 |

| Model | *p*-value | adj. *R^2^* | ΔAICc | w_i_ | K |
| --- | --- | --- | --- | --- | --- |
| DBH+canopy_height | 0.0485 | 0.07 | 10.27 | 0.00 | 4 |
| DBH+tree density | 0.0543 | 0.07 | 10.51 | 0.00 | 4 |
| arthropod total+PCA | 0.0755 | 0.06 | 11.21 | 0.00 | 4 |
| DBH | 0.1146 | 0.03 | 11.74 | 0.00 | 3 |
| null | n/a | n/a | 12.11 | 0.00 | 2 |
| foliage dense >15 | 0.1818 | 0.01 | 12.48 | 0.00 | 3 |
| arthropod total+canopy_height+canopy closure+DBH | 0.0893 | 0.08 | 12.91 | 0.00 | 6 |
| tree density | 0.2543 | 0.01 | 12.98 | 0.00 | 3 |
| arthropod total*PCA+arthropod total+PCA | 0.1634 | 0.04 | 13.62 | 0.00 | 5 |
| canopy closure | 0.4272 | 0.00 | 13.68 | 0.00 | 3 |
| arthropod total | 0.9637 | 0.00 | 14.34 | 0.00 | 3 |
| arthropod total+canopy closure | 0.7247 | 0.00 | 15.99 | 0.00 | 4 |
| arthropod total*DBH+arthropod total+DBH | 0.4813 | 0.00 | 16.45 | 0.00 | 5 |
| arthropod total*canopy closure+DBH+arthropod total+canopy closure | 0.6353 | 0.00 | 18.85 | 0.00 | 6 |
| arthropod total+canopy closure*DBH+canopy closure+DBH | 0.6553 | 0.00 | 18.97 | 0.00 | 6 |
