# Supplementary material for: Do Birds Select Habitat or Food Resources? Nearctic-Neotropic Migrants in Northeastern Costa Rica
Source: PLoS One. 2014 Jan 28;9(1):e86221. doi: 10.1371/journal.pone.0086221 (PMC3904878; doi:10.1371/journal.pone.0086221)
Supplement: Table S3 — Eastern Wood-Pewee habitat use model results. Birds were captured in Tortuguero, Costa Rica, during the 2008 fall migration. The response variable is birds captured per 100 net hours. (DOCX) [file pone.0086221.s010.docx]

Table S3.

| Model | *p*-value | adj. *R^2^* | ΔAICc | w_i_ | K |
| --- | --- | --- | --- | --- | --- |
| PCA | 0.0002 | 0.22 | 0.00 | 0.36 | 3 |
| canopy height | 0.0005 | 0.19 | 2.02 | 0.13 | 3 |
| arthropod winged +PCA | 0.0009 | 0.20 | 2.28 | 0.12 | 4 |
| arthropod total+PCA | 0.0009 | 0.20 | 2.29 | 0.12 | 4 |
| arthropod total+canopy height | 0.0024 | 0.17 | 4.31 | 0.04 | 4 |
| canopy closure+foliage density 0-3m+canopy height | 0.0030 | 0.19 | 4.65 | 0.04 | 5 |
| canopy height+canopy closure+foliage density 0-3m | 0.0030 | 0.19 | 4.65 | 0.04 | 5 |
| canopy closure+foliage density 0-3m | 0.0033 | 0.16 | 4.99 | 0.03 | 4 |
| foliage density 0-3m | 0.0034 | 0.13 | 5.76 | 0.02 | 3 |
| canopy closure+foliage density 0-3m+foliage density 3-15m+canopy height | 0.0053 | 0.19 | 6.14 | 0.02 | 6 |
| arthropod winged+canopy height+canopy closure | 0.0076 | 0.16 | 6.73 | 0.01 | 5 |
| canopy closure+canopy height+foliage density 0-3m+  canopy height*canopy closure | 0.0072 | 0.18 | 6.89 | 0.01 | 6 |
| arthropod winged+canopy height+canopy closure+foliage density 0-3m | 0.0078 | 0.17 | 7.07 | 0.01 | 6 |
| canopy height+canopy closure+foliage density 0-3m+DBH | 0.0079 | 0.17 | 7.11 | 0.01 | 6 |
| canopy closure+foliage density 0-3m+foliage density 3-15m | 0.0098 | 0.15 | 7.34 | 0.01 | 5 |

| Model | *p*-value | adj. *R^2^* | ΔAICc | w_i_ | K |
| --- | --- | --- | --- | --- | --- |
| arthropod winged+canopy closure+foliage density 0-3m | 0.0099 | 0.15 | 7.36 | 0.01 | 5 |
| canopy height+canopy closure+foliage density 0-3m+foliage  density 3-15m +foliage density >15 | 0.0074 | 0.19 | 7.41 | 0.01 | 7 |
|  |  |  |  |  |  |
| foliage density 0-3m+foliage density 3-15m | 0.0134 | 0.12 | 7.96 | 0.01 | 4 |
| arthropod winged+canopy closure+foliage density 0-3m+foliage  density 3-15m | 0.0231 | 0.13 | 9.79 | 0.00 | 6 |
| tree density | 0.0402 | 0.06 | 10.34 | 0.00 | 3 |
| DBH | 0.0522 | 0.05 | 10.80 | 0.00 | 3 |
| arthropod total+DBH*canopy closure+foliage density 0-3m+DBH+  canopy closure | 0.0409 | 0.12 | 12.01 | 0.00 | 7 |
| arthropod total+DBH+canopy closure | 0.0874 | 0.07 | 12.49 | 0.00 | 5 |
| null | n/a | n/a | 12.51 | 0.00 | 2 |
| arthropod total+DBH | 0.1485 | 0.03 | 13.04 | 0.00 | 4 |
| arthropod winged+DBH | 0.1512 | 0.03 | 13.07 | 0.00 | 4 |
| arthropod total | 0.7698 | 0.00 | 14.65 | 0.00 | 3 |
| arthropod winged | 0.9173 | 0.00 | 14.73 | 0.00 | 3 |
| arthropod total*DBH+canopy+closure+arthropod total+DBH | 0.1652 | 0.05 | 15.00 | 0.00 | 7 |
| arthropod total*DBH+arthropod total+DBH | 0.2864 | 0.02 | 15.45 | 0.00 | 5 |
