# Supplementary material for: Do Birds Select Habitat or Food Resources? Nearctic-Neotropic Migrants in Northeastern Costa Rica
Source: PLoS One. 2014 Jan 28;9(1):e86221. doi: 10.1371/journal.pone.0086221 (PMC3904878; doi:10.1371/journal.pone.0086221)
Supplement: Table S4 — Gray-cheeked Thrush habitat use model results. Birds were captured in Tortuguero, Costa Rica, during the 2008 fall migration. The response variable is birds captured per 100 net hours. (DOCX) [file pone.0086221.s011.docx]

Table S4.

| Model | *p*-value | adj. *R^2^* | ΔAICc | w_i_ | K |
| --- | --- | --- | --- | --- | --- |
| ripe fruit+DBH | 0.0177 | 0.11 | 0.00 | 0.38 | 4 |
| canopy closure+foliage density 0-3m+DBH | 0.0591 | 0.08 | 3.01 | 0.08 | 5 |
| null | n/a | n/a | 3.97 | 0.05 | 2 |
| canopy closure | 0.1708 | 0.02 | 4.24 | 0.05 | 3 |
| PCA | 0.1820 | 0.01 | 4.34 | 0.04 | 3 |
| arthropod total*sugar+DBH+arthropod total+sugar | 0.0813 | 0.08 | 4.52 | 0.04 | 6 |
| sugar+PCA | 0.1715 | 0.03 | 4.80 | 0.03 | 4 |
| canopy height | 0.2531 | 0.01 | 4.84 | 0.03 | 3 |
| ripe fruit+PCA | 0.1827 | 0.00 | 4.94 | 0.03 | 4 |
| ripe fruit | 0.2712 | 0.00 | 4.94 | 0.03 | 3 |
| sugar | 0.2831 | 0.00 | 5.00 | 0.03 | 3 |
| tree density | 0.3283 | 0.00 | 5.20 | 0.03 | 3 |
| ripe fruit+canopy closure | 0.2451 | 0.02 | 5.56 | 0.02 | 4 |
| foliage density 3-15m | 0.6287 | 0.00 | 5.96 | 0.02 | 3 |
| foliage density 0-3m | 0.8871 | 0.00 | 6.18 | 0.02 | 3 |

| Model | *p*-value | adj. *R^2^* | ΔAICc | w_i_ | K |
| --- | --- | --- | --- | --- | --- |
| arthropod total | 0.9463 | 0.00 | 6.20 | 0.02 | 3 |
| arthropod total+PCA | 0.4111 | 0.00 | 6.65 | 0.01 | 4 |
| sugar+PCA+sugar*PCA | 0.3059 | 0.01 | 7.08 | 0.01 | 5 |
| ripe fruit+foliage density 0-3m | 0.5156 | 0.00 | 7.13 | 0.01 | 4 |
| arthropod total+ripe fruit | 0.5469 | 0.00 | 7.25 | 0.01 | 4 |
| arthropod total+sugar | 0.5630 | 0.00 | 7.31 | 0.01 | 4 |
| sugar+canopy closure+foliage density 0-3m | 0.4123 | 0.00 | 7.88 | 0.01 | 5 |
| ripe fruit+canopy closure+foliage density 0-3m | 0.4214 | 0.00 | 7.94 | 0.01 | 5 |
| canopy height+canopy closure+foliage density 0-3m | 0.4675 | 0.00 | 8.23 | 0.01 | 5 |
| arthropod total*ripe fruit+arthropod total+ripe fruit | 0.5334 | 0.00 | 8.60 | 0.01 | 5 |
| arthropod total*ripe fruit+PCA+arthropod total+ripe-fruit | 0.3815 | 0.00 | 8.95 | 0.00 | 6 |
| arthropod total*sugar+arthropod total+sugar | 0.6282 | 0.00 | 9.09 | 0.00 | 5 |
| sugar+canopy height+canopy closure+foliage density 0-3m | 0.5005 | 0.00 | 9.85 | 0.00 | 6 |
| ripe fruit+canopy closure+foliage density 0-3m+canopy height | 0.5134 | 0.00 | 9.93 | 0.00 | 6 |
| canopy height+canopy closure+foliage density 0-3m+foliage density 3-15m | 0.6123 | 0.00 | 10.57 | 0.00 | 6 |
