# Supplementary material for: Do Birds Select Habitat or Food Resources? Nearctic-Neotropic Migrants in Northeastern Costa Rica
Source: PLoS One. 2014 Jan 28;9(1):e86221. doi: 10.1371/journal.pone.0086221 (PMC3904878; doi:10.1371/journal.pone.0086221)
Supplement: Table S5 — Mourning Warbler habitat use model results. Birds were captured in Tortuguero, Costa Rica, during the 2008 fall migration. The response variable is birds captured per 100 net hours. (DOCX) [file pone.0086221.s012.docx]

Table S5.

| Model | *p*-value | adj. *R^2^* | ΔAICc | w_i_ | K |
| --- | --- | --- | --- | --- | --- |
| canopy closure+foliage density 0-3m+foliage density 0-3m*canopy closure | 0.0001 | 0.33 | 0.00 | 0.81 | 5 |
| foliage density 0-3m+foliage density 3-15m | 0.0002 | 0.24 | 5.48 | 0.05 | 4 |
| canopy closure+foliage density 0-3m+foliage density 3-15m | 0.0003 | 0.26 | 5.59 | 0.05 | 5 |
| foliage density 0-3m+foliage density 3-15m+DBH | 0.0005 | 0.24 | 6.71 | 0.03 | 5 |
| canopy closure+foliage density 0-3m | 0.0007 | 0.21 | 7.78 | 0.02 | 4 |
| foliage density 0-3m | 0.0005 | 0.19 | 7.98 | 0.01 | 3 |
| arthropod total+foliage density 0-3m | 0.0011 | 0.20 | 8.71 | 0.01 | 4 |
| canopy closure+foliage density 0-3m+canopy height | 0.0018 | 0.20 | 9.53 | 0.01 | 5 |
| foliage density 0-3m+tree density | 0.0021 | 0.18 | 10.05 | 0.01 | 4 |
| DBH+foliage density 0-3m | 0.0023 | 0.18 | 10.23 | 0.00 | 4 |
| foliage density 3-15m | 0.0290 | 0.07 | 15.78 | 0.00 | 3 |
| canopy height+canopy closure | 0.0663 | 0.06 | 17.36 | 0.00 | 4 |
| null | n/a | n/a | 18.53 | 0.00 | 2 |
| PCA | 0.1583 | 0.02 | 18.68 | 0.00 | 3 |
| canopy height | 0.1626 | 0.02 | 18.73 | 0.00 | 3 |

| Model | *p*-value | adj. *R^2^* | ΔAICc | w_i_ | K |
| --- | --- | --- | --- | --- | --- |
| foliage density >15m | 0.2439 | 0.01 | 19.35 | 0.00 | 3 |
| arthropod total | 0.4422 | 0.00 | 20.15 | 0.00 | 3 |
| DBH | 0.4645 | 0.00 | 20.21 | 0.00 | 3 |
| arthropod total+PCA | 0.2862 | 0.01 | 20.45 | 0.00 | 4 |
| arthropod total+canopy height | 0.2928 | 0.01 | 20.49 | 0.00 | 4 |
| tree density | 0.6717 | 0.00 | 20.58 | 0.00 | 3 |
| canopy closure | 0.8026 | 0.00 | 20.70 | 0.00 | 3 |
| DBH+canopy height | 0.3720 | 0.00 | 21.00 | 0.00 | 4 |
| arthropod total+canopy height+canopy closure+DBH | 0.1767 | 0.04 | 21.22 | 0.00 | 6 |
| DBH+tree density | 0.5932 | 0.00 | 21.99 | 0.00 | 4 |
| arthropod total*PCA+arthropod total+PCA | 0.3677 | 0.00 | 22.14 | 0.00 | 5 |
| arthropod total+canopy closure | 0.7038 | 0.00 | 22.35 | 0.00 | 4 |
| arthropod total*DBH+arthropod total+DBH | 0.7656 | 0.00 | 24.28 | 0.00 | 5 |
| arthropod total+canopy closure*DBH+canopy closure+DBH | 0.7308 | 0.00 | 25.84 | 0.00 | 6 |
| arthropod total*canopy closure+DBH+arthropod total+canopy closure | 0.7347 | 0.00 | 25.86 | 0.00 | 6 |
