# Supplementary material for: Do Birds Select Habitat or Food Resources? Nearctic-Neotropic Migrants in Northeastern Costa Rica
Source: PLoS One. 2014 Jan 28;9(1):e86221. doi: 10.1371/journal.pone.0086221 (PMC3904878; doi:10.1371/journal.pone.0086221)
Supplement: Table S6 — Northern Waterthrush habitat use model results. Birds were captured in Tortuguero, Costa Rica, during the 2008 fall migration. The response variable is birds captured per 100 net hours. (DOCX) [file pone.0086221.s013.docx]

Table S6.

| Model | *p*-value | adj. *R^2^* | ΔAICc | w_i_ | K |
| --- | --- | --- | --- | --- | --- |
| arthropod total | 0.0625 | 0.05 | 0.00 | 0.08 | 3 |
| canopy height+canopy closure | 0.0614 | 0.07 | 0.06 | 0.08 | 4 |
| foliage density 3-15m | 0.0752 | 0.04 | 0.32 | 0.07 | 3 |
| arthropod total+foliage density 0-3m | 0.0868 | 0.05 | 0.79 | 0.06 | 4 |
| foliage density 0-3m+foliage density 3-15m | 0.0969 | 0.05 | 1.02 | 0.05 | 4 |
| canopy closure+foliage density 0-3m+foliage density 3-15m | 0.0809 | 0.07 | 1.19 | 0.05 | 5 |
| null | n/a | n/a | 1.40 | 0.04 | 2 |
| arthropod total+canopy height+canopy closure+DBH | 0.0699 | 0.09 | 1.54 | 0.04 | 6 |
| canopy closure+foliage density 0-3m+canopy height | 0.0954 | 0.06 | 1.59 | 0.04 | 5 |
| canopy closure+foliage density 0-3m | 0.1273 | 0.04 | 1.60 | 0.04 | 4 |
| arthropod total+canopy height | 0.1277 | 0.04 | 1.60 | 0.04 | 4 |
| canopy closure+foliage density 0-3m+foliage density 0-3m*canopy closure | 0.0973 | 0.06 | 1.63 | 0.04 | 5 |
| arthropod total+canopy closure | 0.1300 | 0.04 | 1.64 | 0.04 | 4 |
| foliage density 0-3m | 0.1777 | 0.02 | 1.73 | 0.04 | 3 |
| arthropod total+PCA | 0.1438 | 0.04 | 1.86 | 0.03 | 4 |

| Model | *p*-value | adj. *R^2^* | ΔAICc | w_i_ | K |
| --- | --- | --- | --- | --- | --- |
| tree density | 0.2283 | 0.00 | 2.11 | 0.03 | 3 |
| arthropod total*DBH+arthropod total+DBH | 0.1263 | 0.05 | 2.27 | 0.03 | 5 |
| arthropod total*canopy closure+DBH+arthropod total+canopy closure | 0.0973 | 0.07 | 2.43 | 0.03 | 6 |
| foliage density 0-3m+tree density | 0.1950 | 0.02 | 2.50 | 0.02 | 4 |
| canopy closure | 0.3212 | 0.00 | 2.60 | 0.02 | 3 |
| DBH | 0.4297 | 0.00 | 2.98 | 0.02 | 3 |
| canopy height | 0.4659 | 0.00 | 3.08 | 0.02 | 3 |
| arthropod total+canopy closure*DBH+canopy closure+DBH | 0.1317 | 0.06 | 3.26 | 0.02 | 6 |
| PCA | 0.5620 | 0.00 | 3.28 | 0.02 | 3 |
| foliage density 0-3m+foliage density 3-15m+DBH | 0.2000 | 0.03 | 3.42 | 0.02 | 5 |
| foliage density >15 | 0.7316 | 0.00 | 3.51 | 0.01 | 3 |
| DBH+foliage density 0-3m | 0.3721 | 0.00 | 3.87 | 0.01 | 4 |
| DBH+tree density | 0.4484 | 0.00 | 4.26 | 0.01 | 4 |
| arthropod total*PCA+arthropod total+PCA | 0.2783 | 0.02 | 4.26 | 0.01 | 5 |
| DBH+canopy height | 0.7044 | 0.00 | 5.21 | 0.01 | 4 |
