# Supplementary material for: Do Birds Select Habitat or Food Resources? Nearctic-Neotropic Migrants in Northeastern Costa Rica
Source: PLoS One. 2014 Jan 28;9(1):e86221. doi: 10.1371/journal.pone.0086221 (PMC3904878; doi:10.1371/journal.pone.0086221)
Supplement: Table S7 — Prothonotary Warbler habitat use model results. Birds were captured in Tortuguero, Costa Rica, during the 2008 fall migration. The response variable is birds captured per 100 net hours. (DOCX) [file pone.0086221.s014.docx]

Table S7.

| Model | *p*-value | adj. *R^2^* | ΔAICc | w_i_ | K |
| --- | --- | --- | --- | --- | --- |
| sugar+PCA+sugar*PCA | 0.0001 | 0.30 | 0.00 | 0.53 | 5 |
| sugar+PCA | 0.0002 | 0.25 | 2.38 | 0.16 | 4 |
| ripe fruit+PCA | 0.0004 | 0.23 | 4.06 | 0.07 | 4 |
| sugar+canopy closure+foliage density 0-3m | 0.0007 | 0.24 | 4.97 | 0.04 | 5 |
| PCA | 0.0005 | 0.19 | 5.78 | 0.03 | 3 |
| canopy height | 0.0006 | 0.18 | 6.07 | 0.03 | 3 |
| sugar+canopy height+canopy closure+foliage density 0-3m | 0.0013 | 0.24 | 6.39 | 0.02 | 6 |
| ripe fruit+canopy closure | 0.0013 | 0.19 | 6.65 | 0.02 | 4 |
| ripe fruit+canopy closure+foliage density 0-3m | 0.0015 | 0.21 | 6.73 | 0.02 | 5 |
| arthropod total+PCA | 0.0016 | 0.19 | 7.16 | 0.01 | 4 |
| arthropod total*ripe fruit+PCA+arthropod total+ripe-fruit | 0.0019 | 0.22 | 7.30 | 0.01 | 6 |
| canopy height+canopy closure+foliage density 0-3m | 0.0024 | 0.20 | 7.77 | 0.01 | 5 |
| ripe fruit+canopy closure+foliage density 0-3m+canopy height | 0.0025 | 0.21 | 7.97 | 0.01 | 6 |
| foliage density 0-3m | 0.0020 | 0.15 | 8.37 | 0.01 | 3 |
| canopy closure+foliage density 0-3m+DBH | 0.0042 | 0.18 | 9.02 | 0.01 | 5 |

| Model | *p*-value | adj. *R^2^* | ΔAICc | w_i_ | K |
| --- | --- | --- | --- | --- | --- |
| canopy height+canopy closure+foliage density 0-3m+foliage density 3-15m | 0.0043 | 0.20 | 9.25 | 0.01 | 6 |
| ripe fruit+foliage density 0-3m | 0.0052 | 0.15 | 9.60 | 0.00 | 4 |
| sugar | 0.0086 | 0.10 | 11.16 | 0.00 | 3 |
| canopy closure | 0.0096 | 0.10 | 11.36 | 0.00 | 3 |
| arthropod total+sugar | 0.0266 | 0.10 | 13.04 | 0.00 | 4 |
| ripe fruit | 0.0328 | 0.06 | 13.62 | 0.00 | 3 |
| ripe fruit+DBH | 0.0603 | 0.07 | 14.77 | 0.00 | 4 |
| arthropod total*sugar+arthropod total+sugar | 0.0521 | 0.09 | 14.88 | 0.00 | 5 |
| arthropod total+ripe fruit | 0.0816 | 0.06 | 15.41 | 0.00 | 4 |
| arthropod total*sugar+DBH+arthropod total+sugar | 0.0660 | 0.09 | 16.14 | 0.00 | 6 |
| null | n/a | n/a | 16.15 | 0.00 | 2 |
| arthropod total*ripe fruit+arthropod total+ripe fruit | 0.1389 | 0.05 | 17.26 | 0.00 | 5 |
| arthropod total | 0.3331 | 0.00 | 17.41 | 0.00 | 3 |
| foliage density 3-15m | 0.8341 | 0.00 | 18.34 | 0.00 | 3 |
| tree density | 0.8908 | 0.00 | 18.37 | 0.00 | 3 |
