# Supplementary material for: Do Birds Select Habitat or Food Resources? Nearctic-Neotropic Migrants in Northeastern Costa Rica
Source: PLoS One. 2014 Jan 28;9(1):e86221. doi: 10.1371/journal.pone.0086221 (PMC3904878; doi:10.1371/journal.pone.0086221)
Supplement: Table S8 — Red-eyed Vireo habitat use model results. Birds were captured in Tortuguero, Costa Rica, during the 2008 fall migration. The response variable is birds captured per 100 net hours. (DOCX) [file pone.0086221.s015.docx]

Table S8.

| Model | *p*-value | adj. *R^2^* | ΔAICc | w_i_ | K |
| --- | --- | --- | --- | --- | --- |
| foliage density 0-3m | 0.0058 | 0.12 | 0.00 | 0.21 | 3 |
| ripe fruit+foliage density 0-3m | 0.0118 | 0.12 | 0.91 | 0.13 | 4 |
| arthropod total+sugar | 0.0263 | 0.10 | 2.60 | 0.06 | 4 |
| arthropod total+ripe fruit | 0.0302 | 0.09 | 2.89 | 0.05 | 4 |
| sugar | 0.0283 | 0.07 | 2.93 | 0.05 | 3 |
| ripe fruit | 0.0321 | 0.07 | 3.15 | 0.04 | 3 |
| sugar+canopy closure+foliage density 0-3m | 0.0320 | 0.11 | 3.30 | 0.04 | 5 |
| ripe fruit+canopy closure+foliage density 0-3m | 0.0321 | 0.11 | 3.31 | 0.04 | 5 |
| ripe fruit+PCA | 0.0418 | 0.08 | 3.58 | 0.03 | 4 |
| sugar+PCA | 0.0431 | 0.08 | 3.64 | 0.03 | 4 |
| arthropod total*sugar+arthropod total+sugar | 0.0379 | 0.10 | 3.70 | 0.03 | 5 |
| sugar+PCA+sugar*PCA | 0.0419 | 0.10 | 3.95 | 0.03 | 5 |
| canopy height+canopy closure+foliage density 0-3m | 0.0454 | 0.09 | 4.13 | 0.03 | 5 |
| canopy closure+foliage density 0-3m+DBH | 0.0553 | 0.08 | 4.60 | 0.02 | 5 |
| ripe fruit+canopy closure | 0.0686 | 0.06 | 4.62 | 0.02 | 4 |

| Model | *p*-value | adj. *R^2^* | ΔAICc | w_i_ | K |
| --- | --- | --- | --- | --- | --- |
| ripe fruit+DBH | 0.0706 | 0.06 | 4.68 | 0.02 | 4 |
| arthropod total*ripe fruit+arthropod total+ripe fruit | 0.0574 | 0.08 | 4.69 | 0.02 | 5 |
| canopy height | 0.0838 | 0.04 | 4.83 | 0.02 | 3 |
| arthropod total*ripe fruit+PCA+arthropod total+ripe-fruit | 0.0489 | 0.10 | 4.93 | 0.02 | 5 |
| arthropod total*sugar+DBH+arthropod total+sugar | 0.0544 | 0.10 | 5.21 | 0.02 | 6 |
| ripe fruit+canopy closure+foliage density 0-3m+canopy height | 0.0604 | 0.09 | 5.49 | 0.01 | 6 |
| sugar+canopy height+canopy closure+foliage density 0-3m | 0.0606 | 0.09 | 5.50 | 0.01 | 6 |
| PCA | 0.1264 | 0.02 | 5.52 | 0.01 | 3 |
| null | n/a | n/a | 5.73 | 0.01 | 2 |
| arthropod total+PCA | 0.1409 | 0.04 | 6.14 | 0.01 | 4 |
| arthropod total | 0.2345 | 0.01 | 6.48 | 0.01 | 3 |
| canopy height+canopy closure+foliage density 0-3m+foliage density 3-15m | 0.0917 | 0.08 | 6.60 | 0.01 | 6 |
| canopy closure | 0.5206 | 0.00 | 7.53 | 0.00 | 3 |
| tree density | 0.5494 | 0.00 | 7.59 | 0.00 | 3 |
| foliage density 3-15m | 0.7578 | 0.00 | 7.86 | 0.00 | 3 |
