# Supplementary material for: Do Birds Select Habitat or Food Resources? Nearctic-Neotropic Migrants in Northeastern Costa Rica
Source: PLoS One. 2014 Jan 28;9(1):e86221. doi: 10.1371/journal.pone.0086221 (PMC3904878; doi:10.1371/journal.pone.0086221)
Supplement: Table S9 — Swainson's Thrush habitat use model results. Birds were captured in Tortuguero, Costa Rica, during the 2008 fall migration. The response variable is birds captured per 100 net hours. (DOCX) [file pone.0086221.s016.docx]

Table S9.

| Model | *p*-value | adj. *R^2^* | ΔAICc | w_i_ | K |
| --- | --- | --- | --- | --- | --- |
| sugar | 0.0001 | 0.59 | 0.00 | 0.23 | 3 |
| ripe fruit | 0.0001 | 0.57 | 1.83 | 0.09 | 3 |
| arthropod total+sugar | 0.0001 | 0.58 | 1.84 | 0.09 | 4 |
| PCA+ripe fruit+PCA*ripe fruit | 0.0001 | 0.59 | 1.88 | 0.09 | 5 |
| sugar+PCA | 0.0001 | 0.58 | 2.15 | 0.08 | 4 |
| ripe fruit+foliage density 0-3m | 0.0001 | 0.58 | 2.16 | 0.08 | 4 |
| sugar+PCA+sugar*PCA | 0.0001 | 0.59 | 2.68 | 0.06 | 5 |
| sugar+canopy closure+foliage density 0-3m | 0.0001 | 0.58 | 3.13 | 0.05 | 5 |
| ripe fruit+DBH | 0.0001 | 0.57 | 3.34 | 0.04 | 4 |
| ripe fruit+PCA | 0.0001 | 0.57 | 3.42 | 0.04 | 4 |
| arthropod total+ripe fruit | 0.0001 | 0.57 | 3.61 | 0.04 | 4 |
| ripe fruit+canopy closure | 0.0001 | 0.57 | 3.90 | 0.03 | 4 |
| tree density+arthropod total+sugar | 0.0001 | 0.57 | 4.24 | 0.03 | 5 |
| ripe fruit+canopy closure+foliage density 0-3m | 0.0001 | 0.57 | 4.57 | 0.02 | 5 |
| PCA+arthropod total+ripe-fruit | 0.0001 | 0.57 | 5.31 | 0.02 | 5 |

| Model | *p*-value | adj. *R^2^* | ΔAICc | w_i_ | K |
| --- | --- | --- | --- | --- | --- |
| sugar+canopy height+canopy closure+foliage density 0-3m | 0.0001 | 0.58 | 5.34 | 0.02 | 6 |
| ripe fruit+canopy closure+foliage density 0-3m+canopy height | 0.0001 | 0.57 | 6.90 | 0.01 | 6 |
| foliage density 0-3m | 0.0009 | 0.17 | 39.05 | 0.00 | 3 |
| canopy closure+foliage density 0-3m+DBH | 0.0031 | 0.19 | 40.52 | 0.00 | 5 |
| canopy height+canopy closure+foliage density 0-3m | 0.0043 | 0.18 | 41.25 | 0.00 | 5 |
| canopy height+canopy closure+foliage density 0-3m+foliage density 3-15m | 0.0105 | 0.16 | 43.61 | 0.00 | 6 |
| null | n/a | n/a | 48.32 | 0.00 | 2 |
| arthropod total | 0.2121 | 0.01 | 48.93 | 0.00 | 3 |
| PCA | 0.2367 | 0.01 | 49.09 | 0.00 | 3 |
| canopy height | 0.2480 | 0.01 | 49.16 | 0.00 | 3 |
| arthropod total+PCA | 0.0000 | 0.02 | 49.85 | 0.00 | 4 |
| foliage density 3-15m | 0.4216 | 0.00 | 49.88 | 0.00 | 3 |
| DBH | 0.6979 | 0.00 | 50.40 | 0.00 | 3 |
| canopy closure | 0.7947 | 0.00 | 50.48 | 0.00 | 3 |
| tree density | 0.9372 | 0.00 | 50.55 | 0.00 | 3 |
