# Supplementary material for: Do Birds Select Habitat or Food Resources? Nearctic-Neotropic Migrants in Northeastern Costa Rica
Source: PLoS One. 2014 Jan 28;9(1):e86221. doi: 10.1371/journal.pone.0086221 (PMC3904878; doi:10.1371/journal.pone.0086221)
Supplement: Table S10 — Traill's Flycatcher habitat use model results. Birds were captured in Tortuguero, Costa Rica, during the 2008 fall migration. The response variable is birds captured per 100 net hours. (DOCX) [file pone.0086221.s017.docx]

Table S10.

| Model | *p*-value | adj. *R^2^* | ΔAICc | w_i_ | K |
| --- | --- | --- | --- | --- | --- |
| PCA | 0.0007 | 0.18 | 0.00 | 0.26 | 3 |
| arthropod winged+PCA | 0.0020 | 0.18 | 1.09 | 0.15 | 4 |
| sugar+PCA | 0.0031 | 0.17 | 1.99 | 0.09 | 4 |
| ripe fruit+PCA | 0.0031 | 0.17 | 2.00 | 0.09 | 4 |
| foliage density 0-3m | 0.0034 | 0.13 | 2.93 | 0.06 | 3 |
| arthropod total+ripe fruit+PCA | 0.0051 | 0.17 | 3.00 | 0.06 | 5 |
| PCA+sugar+PCA*sugar | 0.0055 | 0.17 | 3.15 | 0.05 | 5 |
| canopy height+canopy closure+foliage density 0-3m | 0.0070 | 0.16 | 3.70 | 0.04 | 5 |
| canopy height+canopy closure | 0.0078 | 0.14 | 3.96 | 0.04 | 4 |
| foliage density 0-3m+foliage densirt 3-15m | 0.0101 | 0.13 | 4.51 | 0.03 | 4 |
| canopy closure+foliage density 0-3m+foliage densirt 3-15m | 0.0106 | 0.15 | 4.66 | 0.03 | 5 |
| arthropod total*ripe fruit+arthropod total+ripe fruit+canopy closure+foliage density 0-3m | 0.0098 | 0.18 | 5.29 | 0.02 | 7 |
|  |  |  |  |  |  |
| sugar+canopy closure+foliage density 0-3m | 0.0159 | 0.13 | 5.60 | 0.02 | 5 |
| ripe fruit+canopy closure+foliage density 0-3m | 0.0159 | 0.13 | 5.60 | 0.02 | 5 |
| canopy closure+foliage density 0-3m+foliage densirt 3-15m+canopy height | 0.0158 | 0.15 | 5.98 | 0.01 | 6 |

| Model | *p*-value | adj. *R^2^* | ΔAICc | w_i_ | K |
| --- | --- | --- | --- | --- | --- |
| sugar+canopy height+canopy closure+foliage density 0-3m | 0.0171 | 0.14 | 6.18 | 0.01 | 5 |
| canopy closure | 0.0310 | 0.15 | 7.02 | 0.01 | 3 |
| DBH*canopy closure+foliage density 0-3m+DBH+canopy closure | 0.0319 | 0.12 | 7.76 | 0.01 | 6 |
| tree density | 0.0498 | 0.05 | 8.00 | 0.00 | 3 |
| null | n/a | n/a | 9.66 | 0.00 | 2 |
| arthropod winged | 0.1934 | 0.01 | 10.12 | 0.00 | 3 |
| arthropod winged+sugar | 0.1905 | 0.03 | 10.71 | 0.00 | 4 |
| sugar | 0.3127 | 0.00 | 10.83 | 0.00 | 3 |
| arthropod total | 0.3919 | 0.00 | 11.13 | 0.00 | 3 |
| arthropod winged+ripe fruit | 0.2327 | 0.02 | 11.14 | 0.00 | 4 |
| ripe fruit | 0.3957 | 0.00 | 11.14 | 0.00 | 3 |
| arthropod total+sugar | 0.3595 | 0.00 | 12.05 | 0.00 | 4 |
| arthropod total+ripe fruit | 0.4296 | 0.00 | 12.43 | 0.00 | 4 |
| arthropod total*ripe fruit+arthropod total+ripe fruit | 0.3760 | 0.00 | 13.32 | 0.00 | 5 |
| arthropod total*sugar+arthropod total+sugar | 0.4298 | 0.00 | 13.69 | 0.00 | 5 |
