# Supplementary material for: Do Birds Select Habitat or Food Resources? Nearctic-Neotropic Migrants in Northeastern Costa Rica
Source: PLoS One. 2014 Jan 28;9(1):e86221. doi: 10.1371/journal.pone.0086221 (PMC3904878; doi:10.1371/journal.pone.0086221)
Supplement: Table S11 — Veery habitat use model results. Birds were captured in Tortuguero, Costa Rica, during the 2008 fall migration. The response variable is birds captured per 100 net hours. (DOCX) [file pone.0086221.s018.docx]

Table S11.

| Model | *p*-value | adj. *R^2^* | ΔAICc | w_i_ | K |
| --- | --- | --- | --- | --- | --- |
| ripe fruit+DBH | 0.0040 | 0.16 | 0.00 | 0.39 | 4 |
| tree density+arthropod total | 0.0086 | 0.17 | 1.93 | 0.15 | 4 |
| sugar | 0.0168 | 0.08 | 3.38 | 0.07 | 3 |
| ripe fruit | 0.0198 | 0.08 | 3.68 | 0.06 | 3 |
| arthropod total+sugar | 0.0331 | 0.09 | 4.48 | 0.04 | 4 |
| arthropod total+ripe fruit | 0.0375 | 0.08 | 4.74 | 0.04 | 4 |
| arthropod total*ripe fruit+arthropod total+ripe fruit | 0.0404 | 0.10 | 5.26 | 0.03 | 5 |
| sugar+PCA | 0.0514 | 0.07 | 5.41 | 0.03 | 4 |
| arthropod total*sugar+arthropod total+sugar | 0.0448 | 0.09 | 5.50 | 0.02 | 5 |
| ripe fruit+PCA | 0.0629 | 0.07 | 5.84 | 0.02 | 4 |
| canopy closure+foliage density 0-3m+DBH | 0.0533 | 0.09 | 5.91 | 0.02 | 5 |
| ripe fruit+canopy closure | 0.0666 | 0.06 | 5.96 | 0.02 | 4 |
| ripe fruit+foliage density 0-3m | 0.0676 | 0.06 | 5.99 | 0.02 | 4 |
| sugar+PCA+sugar*PCA | 0.0831 | 0.07 | 6.98 | 0.01 | 5 |
| null | n/a | n/a | 7.13 | 0.01 | 2 |

| Model | *p*-value | adj. *R^2^* | ΔAICc | w_i_ | K |
| --- | --- | --- | --- | --- | --- |
| arthropod total | 0.1754 | 0.02 | 7.44 | 0.01 | 3 |
| arthropod total*ripe fruit+PCA+arthropod total+ripe-fruit | 0.0805 | 0.08 | 7.65 | 0.01 | 6 |
| sugar+canopy closure+foliage density 0-3m | 0.1270 | 0.05 | 8.02 | 0.01 | 5 |
| ripe fruit+canopy closure+foliage density 0-3m | 0.1468 | 0.05 | 8.38 | 0.01 | 5 |
| foliage density 0-3m | 0.3838 | 0.00 | 8.57 | 0.01 | 3 |
| canopy height | 0.6536 | 0.00 | 9.15 | 0.00 | 3 |
| foliage density 3-15m | 0.6601 | 0.00 | 9.16 | 0.00 | 3 |
| canopy closure | 0.6733 | 0.00 | 9.18 | 0.00 | 3 |
| tree density | 0.7017 | 0.00 | 9.21 | 0.00 | 3 |
| PCA | 0.9111 | 0.00 | 9.35 | 0.00 | 3 |
| arthropod total+PCA | 0.3975 | 0.00 | 9.74 | 0.00 | 4 |
| sugar+canopy height+canopy closure+foliage density 0-3m | 0.2160 | 0.04 | 10.39 | 0.00 | 6 |
| ripe fruit+canopy closure+foliage density 0-3m+canopy height | 0.2415 | 0.03 | 10.71 | 0.00 | 6 |
| canopy height+canopy closure+foliage density 0-3m | 0.6255 | 0.00 | 12.23 | 0.00 | 5 |
| canopy height+canopy closure+foliage density 0-3m+foliage density 3-15m | 0.6368 | 0.00 | 13.88 | 0.00 | 6 |
