# Supplementary material for: Do Birds Select Habitat or Food Resources? Nearctic-Neotropic Migrants in Northeastern Costa Rica
Source: PLoS One. 2014 Jan 28;9(1):e86221. doi: 10.1371/journal.pone.0086221 (PMC3904878; doi:10.1371/journal.pone.0086221)
Supplement: Table S12 — Linear model variables for 11 migrant species captured in Tortuguero, Costa Rica, and their associated beta estimates, standard errors and 95% confidence intervals for best or averaged models of each study species. Asterisk indicates an averaged model. (DOCX) [file pone.0086221.s019.docx]

Table S12.

| Species and Model Parameters | Estimate | SE | Lower 95% CI | Upper 95% CI |
| --- | --- | --- | --- | --- |
| *Acadian Flycatcher** |  |  |  |  |
| intercept | 0.262 | 0.058 | 0.148 | 0.377 |
| ripe fruit | 0.002 | 0.001 | 0.001 | 0.003 |
| sugar | 0.019 | 0.019 | -0.018 | 0.055 |
| PCA1 | -0.119 | 0.03 | -0.178 | -0.059 |
| *Canada Warbler* |  |  |  |  |
| intercept | -0.562 | 0.27 | -1.104 | -0.02 |
| foliage density 0-3m | 0.947 | 0.281 | 0.383 | 1.51 |
| foliage density 3-15m | 6.001 | 2.533 | 0.921 | 11.082 |
| *Eastern Wood-Pewee* |  |  |  |  |
| intercept | 0.523 | 0.142 | 0.238 | 0.808 |
| PCA1 | -0.318 | 0.079 | -0.476 | -0.16 |
| *Grey-cheeked Thrush* |  |  |  |  |
| intercept | -0.306 | 0.788 | -1.886 | 1.275 |
| ripe fruit | 0.012 | 0.01 | -0.007 | 0.032 |
| DBH | 0.139 | 0.051 | 0.036 | 0.242 |
| *Mourning Warbler* |  |  |  |  |
| intercept | 3.491 | 1.896 | -0.313 | 7.294 |
| canopy closure | -0.039 | 0.021 | -0.08 | 0.002 |
| foliage density 0-3m | -9.044 | 3.102 | -15.268 | -2.819 |
| foliage density 0-3m*canopy closure | 0.109 | 0.034 | 0.041 | 0.177 |
| *Northern Waterthrush** |  |  |  |  |
| intercept | -1.72 | 1.733 | -5.117 | 1.677 |
| arthropod total | 0.023 | 0.013 | -0.002 | 0.049 |
| canopy height | -0.014 | 0.008 | -0.029 | 0.002 |
| canopy closure | 0.021 | 0.015 | -0.008 | 0.05 |
| foliage density 3-15m | 2.139 | 1.231 | -0.274 | 4.551 |
| foliage density 0-3m | -0.452 | 0.608 | -1.645 | 0.74 |
| DBH | -0.001 | 0.002 | -0.005 | 0.002 |
| foliage density 0-3m*canopy closure | 0.008 | 0.005 | -0.002 | 0.018 |
| PCA1 | -0.003 | 0.005 | -0.012 | 0.006 |

| Species and Model Parameters | Estimate | SE | Lower 95% CI | Upper 95% CI |
| --- | --- | --- | --- | --- |
| *Prothonotary Warbler* |  |  |  |  |
| intercept | 0.748 | 0.172 | 0.402 | 1.093 |
| sugar | 0.024 | 0.076 | -0.128 | 0.177 |
| PCA1 | -0.204 | 0.104 | -0.412 | 0.004 |
| sugar*PCA1 | -0.184 | 0.085 | -0.354 | -0.013 |
| *Red-eyed Vireo** |  |  |  |  |
| intercept | 0.062 | 0.032 | 0.06 | 0.073 |
| foliage density 0-3m | 0.179 | 0.07 | -0.042 | 0.315 |
| ripe fruit | 0.003 | 0.002 | 0.001 | 0.007 |
| *Swainson's Thrush** |  |  |  |  |
| intercept | 1.68 | 0.285 | 1.121 | 2.239 |
| ripe fruit | 0.017 | 0.003 | 0.012 | 0.022 |
| arthropod total | 0.005 | 0.007 | -0.019 | 0.009 |
| sugar | 0.123 | 0.014 | 0.095 | 0.151 |
| PCA1 | 0.007 | 0.026 | -0.043 | 0.058 |
| PCA1*ripe fruit | -0.003 | 0.002 | -0.007 | 0.001 |
| *Traill's Flycatcher** |  |  |  |  |
| intercept | 0.425 | 0.293 | -0.149 | 0.999 |
| arthropod winged | 0.013 | 0.009 | -0.006 | 0.031 |
| PCA1 | -0.334 | 0.094 | -0.518 | -0.149 |
| sugar | 0.006 | 0.011 | -0.015 | 0.027 |
| *Veery** |  |  |  |  |
| intercept | 0.346 | 0.323 | -0.288 | 0.979 |
| ripe fruit | 0.006 | 0.002 | 0.001 | 0.011 |
| DBH | 0.03 | 0.012 | 0.006 | 0.054 |
| tree density | -0.15 | 0.383 | -0.901 | 0.601 |
| arthropod total | 0.009 | 0.006 | -0.004 | 0.021 |
